# Thermal Growth of Au–Fe Heterometallic Carbonyl Clusters Containing N-Heterocyclic Carbene and Phosphine Ligands

**DOI:** 10.1021/acs.inorgchem.9b02912

**Published:** 2020-01-31

**Authors:** Beatrice Berti, Marco Bortoluzzi, Cristiana Cesari, Cristina Femoni, Maria Carmela Iapalucci, Rita Mazzoni, Federico Vacca, Stefano Zacchini

**Affiliations:** † Dipartimento di Chimica Industriale “Toso Montanari”, University of Bologna, Viale Risorgimento 4, I-40136 Bologna, Italy; ‡ Dipartimento di Scienze Molecolari e Nanosistemi, Ca’ Foscari University of Venice, Via Torino, 155-30175 Mestre, Venice, Italy

## Abstract

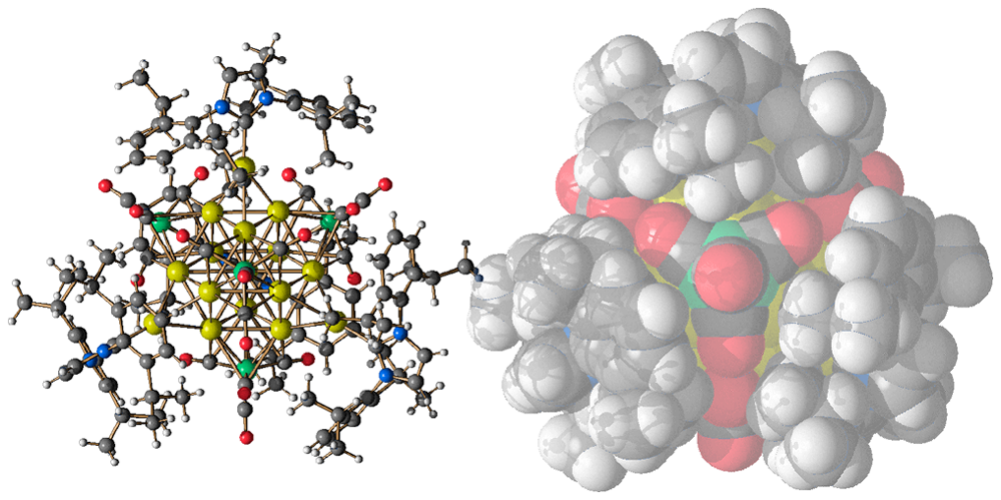

The thermal reactions of [NEt_4_][Fe(CO)_4_(AuNHC)] [NHC = IMes ([NEt_4_][**1**]) or IPr ([NEt_4_][**2**]); IMes = C_3_N_2_H_2_(C_6_H_2_Me_3_)_2_; IPr = C_3_N_2_H_2_(C_6_H_3_^i^Pr_2_)_2_], Fe(CO)_4_(AuNHC)_2_ [NHC = IMes (**3**) or IPr (**4**)], Fe(CO)_4_(AuIMes)(AuIPr) (**5**), and Fe(CO)_4_(AuNHC)(AuPPh_3_) [NHC = IMes (**6**) or IPr (**7**)] were investigated in different solvents [CH_2_Cl_2_, CH_3_CN, dimethylformamide, and dimethyl sulfoxide (dmso)] and at different temperatures (50–160 °C) in an attempt to obtain higher-nuclearity clusters. **1** and **2** completely decomposed in refluxing CH_2_Cl_2_, resulting in [Fe_2_(CO)_8_(AuNHC)]^−^ [NHC = IMes (**10**) or IPr (**11**)]. Traces of [Fe_3_(CO)_10_(CCH_3_)]^−^ (**12**) were obtained as a side product. Conversely, **6** decomposed in refluxing CH_3_CN, affording the new cluster [Au_3_{Fe(CO)_4_}_2_(PPh_3_)_2_]^−^ (**15**). The relative stability of the two isomers found in the solid state structure of **15** was computationally investigated. **4** was very stable, and only after prolonged heating above 150 °C in dmso was limited decomposition observed, affording small amounts of [Fe_3_S(CO)_9_]^2–^ (**9**), [HFe(CO)_4_]^−^ (**16**), and [Au_16_S{Fe(CO)_4_}_4_(IPr)_4_]^*n*+^ (**17**). A dicationic nature for **17** was proposed on the basis of density functional theory calculations. All of the other reactions examined led to species that were previously reported. The molecular structures of the new clusters **11**, **12**, **15**, and **17** were determined by single-crystal X-ray diffraction as their [NEt_4_][**11**]·1.5toluene, [Au(IMes)_2_][**15**]·0.67CH_2_Cl_2_, [NEt_4_][**12**], and [**17**][BF_4_]_*n*_·solvent salts, respectively.

## Abstract 2

The nuclearity of Au−Fe molecular clusters has been increased by thermal methods, resulting in heterometallic species stabilized by CO, NHC, and phosphine ligands.

## 1 Introduction

The Fe(CO)_4_ group was a very versatile fragment for stabilizing gold clusters.^1^ These included low-nuclearity complexes such as Fe(CO)_4_(AuPPh_3_)_2_,^2^ Fe(CO)_4_(AuNHC)_2_ [NHC = IMes, IPr, or IBu; IMes = C_3_N_2_H_2_(C_6_H_2_Me_3_)_2_; IPr = C_3_N_2_H_2_(C_6_H_3_^i^Pr_2_)_2_; IBu = C_3_N_2_H_2_(CMe_3_)_2_],^3^ and [Fe(CO)_4_(AuNHC)]^−^,^4^ as well as one- and two-dimensional gold clusters, such as [Au_3_Fe_2_(CO)_8_(IMes)_2_]^−^,^3^ [Au_3_Fe(CO)_4_(dppm)_2_]^+^ (dppm = Ph_2_PCH_2_PPh_2_),^5^ [Au_3_Fe_2_(CO)_8_(dppm)]^−^,^6^ [Au_3_{Fe(CO)_4_}_3_]^3–^,^7^ [Au_4_{Fe(CO)_4_}_4_]^4–^,^8^ [Au_5_Fe_4_(CO)_16_]^3–^,^9^ [Au_5_Fe_2_(CO)_8_(dppm)_2_]^+^,^6^ and Au_8_Fe_4_(CO)_16_(dppe)_4_ (dppe = Ph_2_PCH_2_CH_2_PPh_2_).^10^ These clusters could be viewed as being composed of [Fe(CO)_4_]^2–^ moieties and Au(I) ions, containing in some cases additional NHC and/or phosphine ligands. The general strategy for their syntheses was the reaction of Colman’s reagent Na_2_[Fe(CO)_4_]·2thf with Au(I) complexes such as Au(Et_2_S)Cl, [AuBr_2_]^−^, Au(PPh_3_)Cl, Au(NHC)Cl, Au_2_(dppm)Cl_2_, and Au_2_(dppe)Cl_2_.

In addition, three-dimensional metalloid Fe–Au clusters were obtained from the redox condensation of [Fe_3_(CO)_11_]^2–^ and [AuCl_4_]^−^. This category included Au–Fe–CO molecular nanoclusters such as [Au_21_Fe_10_(CO)_40_]^5–^, [Au_22_Fe_12_(CO)_48_]^6–^, [Au_28_Fe_14_(CO)_52_]^8–^, and [Au_34_Fe_14_(CO)_50_]^8–^, stabilized by Fe(CO)_4_ and Fe(CO)_3_ groups present on their surface.^9^ The Au atoms within their Au_*n*_ core displayed a formal oxidation state between +1 and 0. Linear Fe–Au–Fe staple motifs, reminiscent of the very well-known S–Au–S staple motifs found in Au–thiolate nanoclusters, were present on the surface of these organometallic Au–Fe carbonyl clusters.^9,11,12^

The desire for atomically precise (molecular) gold nanoclusters has incredibly grown in recent years, because of their fundamental aspects and properties as well as possible applications.^13−19^ Thiolate and phosphine ligands were widely employed for the preparation of molecular gold nanoclusters, but other ligands,^20−22^ including organometallic fragments, might be employed. In this sense, the combination of Fe(CO)_4_, NHC, and phosphine ligands might be an interesting approach for the growth of new gold clusters. In addition, Au–Fe carbonyl clusters were also useful platforms for the study of intramolecular aurophilic interactions.^1,7,11,23−25^ In light of this broad interest in gold clusters and aurophilic interactions, the preparation of gold-containing molecular clusters of increasing sizes is still a fascinating challenge.

**Scheme 1:**
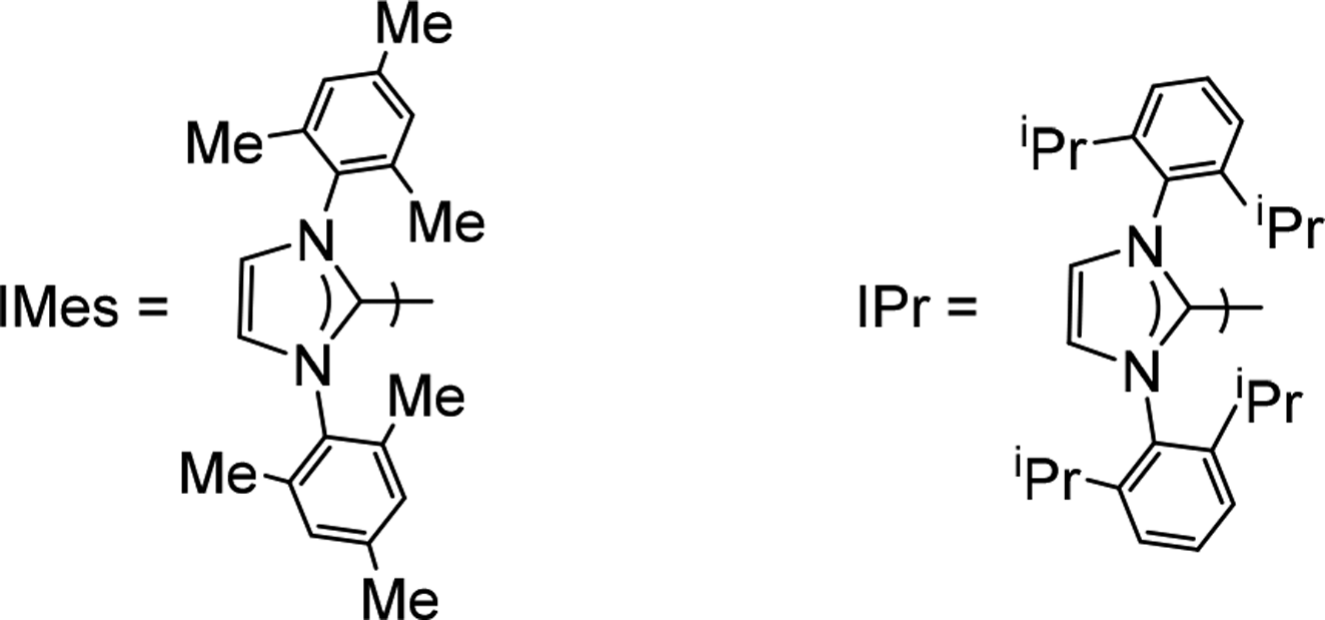
IMes and IPr Ligands

Thermal reactions of low-nuclearity precursors might be an alternative to redox condensation for the preparation of higher-nuclearity Fe–Au carbonyl clusters. Indeed, it was recently reported that the thermal treatment of Fe(CO)_4_(AuIMes)_2_ resulted in [Au_3_{Fe(CO)_4_}_3_]^3–^ or [Au_3_Fe_2_(CO)_8_(IMes)_2_]^−^ depending of the experimental conditions.^3,7^ In both cases, the reactions involved ionization of the neutral precursors and rearrangement of the ligands, with retention of the original −2 and +1 oxidation states for Fe and Au, respectively. It is also worth noting that thermal reaction of Fe(CO)_4_(AuIMes)_2_ was the only synthetic approach viable for the [Au_3_{Fe(CO)_4_}_3_]^3–^ trinuclear compound, whereas the direct reaction of Na_2_[Fe(CO)_4_]·2thf with [AuBr_2_]^−^ afforded selectively the [Au_4_{Fe(CO)_4_}_4_]^4–^ tetranuclear compound.^8^ These preliminary results prompted us to systematically study the thermal reactions of low-nuclearity monoanionic and neutral precursors such as [NEt_4_][Fe(CO)_4_(AuNHC)] [NHC = IMes ([NEt_4_][**1**]) or IPr ([NEt_4_][**2**]); IMes = C_3_N_2_H_2_(C_6_H_2_Me_3_)_2_; IPr = C_3_N_2_H_2_(C_6_H_3_^i^Pr_2_)_2_ (Scheme 1)], Fe(CO)_4_(AuNHC)_2_ [NHC = IMes (**3**) or IPr (**4**)], Fe(CO)_4_(AuIMes)(AuIPr) (**5**), and Fe(CO)_4_(AuNHC)(AuPPh_3_) [NHC = IMes (**6**) or IPr (**7**)]. The outcomes of the different reactions are reported herein, supported by a computational investigation of new products characterized by unusual isomerism and bond structure.

## 2 Results and Discussion

**Scheme 2:**
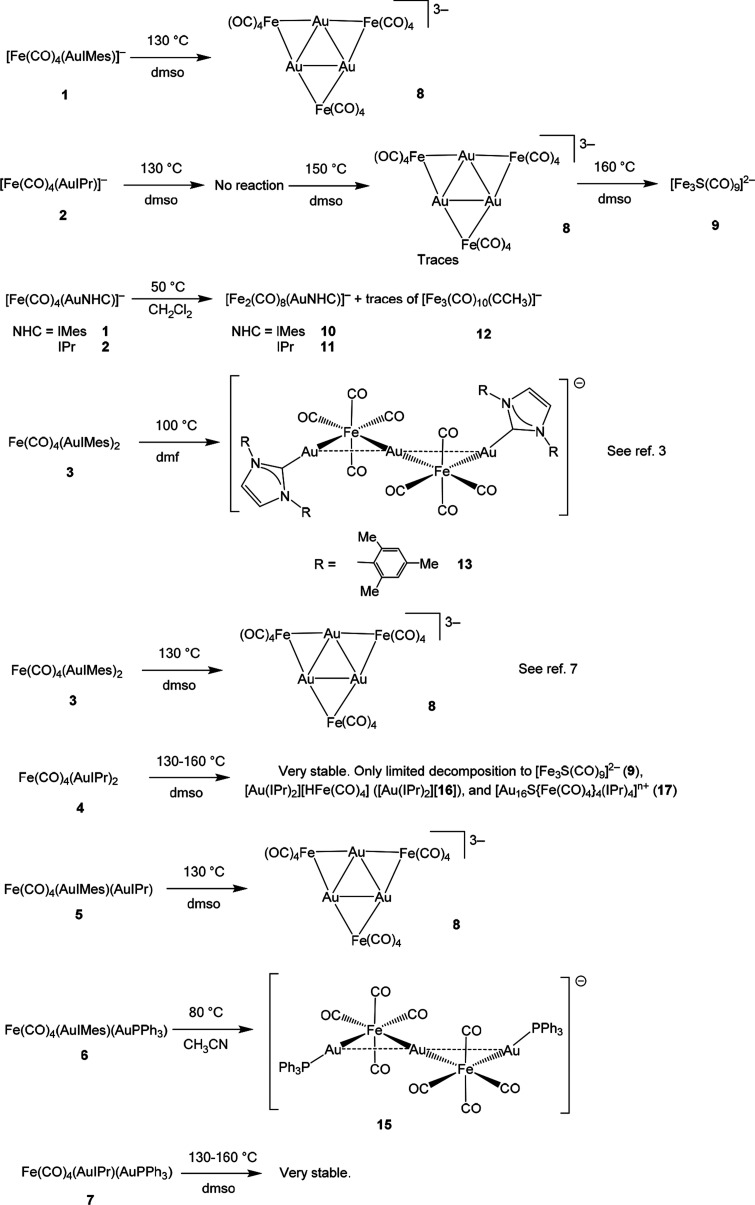
Thermal Reactions of **1–7**

The thermal reactions of complexes **1–7** were investigated with the goal of obtaining higher-nuclearity species. As a general strategy, **1–7** were heated in different solvents [CH_2_Cl_2_, CH_3_CN, dimethylformamide (dmf), and dimethyl sulfoxide (dmso)] at temperatures in the range of 50–160 °C monitoring the evolution of the reactions over time by infrared (IR) spectroscopy in the ν_CO_ region. Anionic compounds were examined as [NEt_4_]^+^ salts. The crude reaction mixtures were recovered after removal of the organic solvent under reduced pressure or, in the case of dmf or dmso as the solvent, by precipitation with H_2_O in the presence of a suitable tetraalkyl-ammonium salt. The solid residue was extracted with solvents of increasing polarity in the attempt to separate the products from the crude reaction mixtures. Further details can be found in the Experimental Section. All of the results obtained are summarized in Scheme 2. The new results herein obtained can be summarized as follows.

(1) Heating monoanions **1** and **2** as [NEt_4_]^+^ salts in CH_2_Cl_2_ at refluxing temperature resulted in the formation of [Fe_2_(CO)_8_(AuNHC)]^−^ [NHC = IMes (**10**) or IPr (**11**)]. Traces of [Fe_3_(CO)_10_(CCH_3_)]^−^ (**12**) were obtained as a side product. These represented an interesting addition to the limited number of compounds with the Fe_2_(CO)_6_(μ-CO)_2_ unit (section 2.1).

(2) The thermal decomposition of **6** in CH_3_CN at 80 °C afforded the larger [Au_3_{Fe(CO)_4_}_2_(PPh_3_)_2_]^−^ (**15**) cluster that was present as two isomers in the solid state structure (section 2.2).

(3) **4** was only partially decomposed after prolonged heating in dmso at 130–160 °C, resulting in a mixture of [Fe_3_S(CO)_9_]^2–^ (**9**), [HFe(CO)_4_]^−^ (**16**), and [Au_16_S{Fe(CO)_4_}_4_(IPr)_4_]^*n*+^ (**17**). Compound **17** was rather interesting because it was a high-nuclearity Au cluster containing an interstitial μ_12_-S atom stabilized on the surface by Fe(CO)_4_ fragments and IPr ligands (section 2.3).

The other reactions studied led to compounds that were previously published, and therefore, they will not be discussed further.^3,4,7^ These included [Au_3_{Fe(CO)_4_}_3_]^3–^ (**8**),^7^ [Fe_3_S(CO)_9_]^2–^ (**9**),^26^ and [Au_3_Fe_2_(CO)_8_(IMes)_2_]^−^ (**13**).^3^ In particular, **8** was obtained as the main product of several reactions. We reported the synthesis of **8** by the thermal decomposition of **3** in dmso at 130 °C in a previous communication.^7^ As summarized in Scheme 2, **8** could also be obtained by thermal treatment of **1**, **2**, and **5**. **13** was obtained by the thermal reaction of **3** at lower temperatures (≤100 °C) in dmf or dmso,^3^ whereas **8** was formed at higher temperatures. The computed Gibbs energy variations (C-PCM/PBEh-3c calculations) for reactions 1 and 2 were −13.1 and −23.1 kcal mol^–1^, respectively, and suggested that the formation of [Au(IMes)_2_]^+^ was the driving force. As a general comment on Scheme 2, IPr-containing species were far more thermally stable than IMes-containing species.
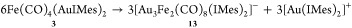
1
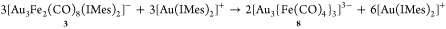
2

### 2.1 Syntheses and Characterization of [Fe_2_(CO)_8_(AuNHC)]^−^ [NHC = IMes (**10**) or IPr (**11**)] and [Fe_3_(CO)_10_(CCH_3_)]^−^ (**12**)

Anionic species **1** and **2** were not stable in chlorinated solvents such as CH_2_Cl_2_ at room temperature. Complete decomposition occurred after heating at 50 °C, resulting in the formation of the new species [Fe_2_(CO)_8_(AuNHC)]^−^ [NHC = IMes (**10**) or IPr (**11**)]. Formation of **10** and **11** required the formal oxidation of iron from −2, as in **1** and **2**, to −1, as in the final products. Because this reaction did not occur in nonchlorinated solvents even after heating for several hours, we could rule out the possibility that adventitious oxygen was the oxidizing species. Thus, the oxidant should be CH_2_Cl_2_ itself.^27^ Unfortunately, all attempts to identify the products of the reduction of CH_2_Cl_2_ by GC-MS analyses failed. Therefore, it was not possible to deduce the mechanism of the reaction.

**Figure 1:**
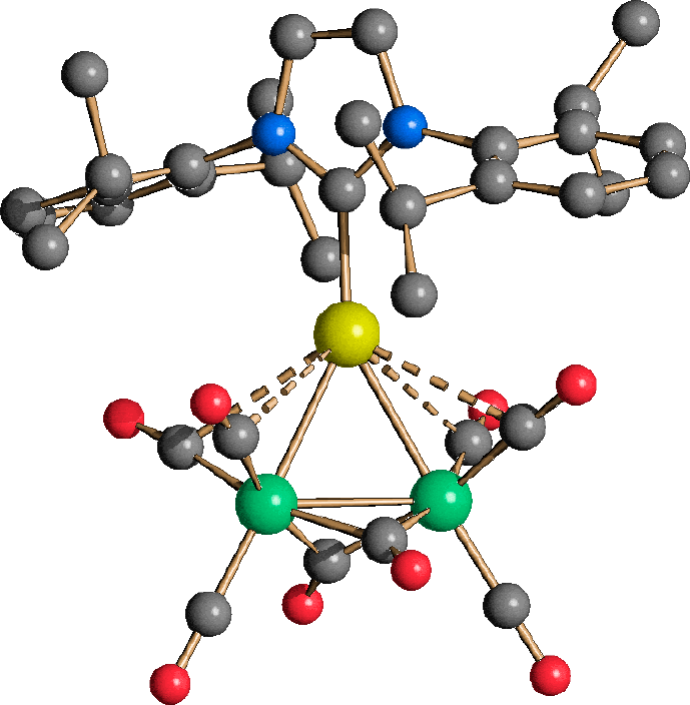
Molecular structure of **11**. Au–C(O) contacts [2.830(19)–2.977(19) Å] are represented as dashed lines. Hydrogen atoms have been omitted for the sake of clarity (green, Fe; yellow, Au; blue, N; red, O; gray, C). Selected bond lengths (angstroms): Fe–Fe, 2.573(4); Fe–Au, 2.665(3) and 2.677(3); Au–C_carbene_, 2.013(18); Fe–C(O)_bridge_, 1.90(2)–1.969(18); Fe–C(O)_terminal_, 1.73(2)–1.83(3).

Compounds **10** and **11** were characterized by means of IR and multinuclear nuclear magnetic resonance (NMR) spectroscopy, and the molecular structure of **11** was crystallographycally determined as its [NEt_4_][**11**]·1.5toluene salt (Figure 1). The molecular structure of **11** may be viewed as the result of the addition of a [AuIPr]^+^ fragment to [Fe_2_(CO)_8_]^2–^. It displayed six terminal and two edge-bridging carbonyl ligands, as previously found in the PPh_3_ derivative [Fe_2_(CO)_8_(AuPPh_3_)]^−^.^28^ Conversely, the related copper species [Fe_2_(CO)_8_(CuPCy_3_)]^−^^29^ displayed only terminal carbonyls. **11** displayed also some short sub-van der Waals Au···C(O) contacts. The structure of **11** was an interesting addition to the limited number of compounds with the Fe_2_(CO)_6_(μ-CO)_2_ unit.^30,31^ The Fe–Fe bond distance of such compounds spanned a very large range (2.39–2.62 Å). In the case of **11**, the Fe–Fe distance [2.573(4) Å] was between those of Fe_2_(CO)_9_ (2.52 Å)^32^ and [Fe_2_(CO)_8_(AuPPh_3_)]^−^ (2.605 Å).^28^

The ^1^H and ^13^C{^1^H} NMR spectra of **11** (Figures S1 and S2) displayed all of the expected resonances due to the IPr group. Conversely, in the carbonyl region of the ^13^C{^1^H} NMR spectra recorded at 298 and 273 K, only a single sharp resonance at 230.5 ppm was detected. Coalescence was, then, observed at 213 K (Figure S3), suggesting the presence of a fluxional behavior that made the eight CO ligands equivalent at higher temperatures. The structures of **10** and **11** were also optimized by means of density functional theory (DFT) calculations. The root-mean-square deviation (RMSD) between the experimental and computed structures of the anion of **11** was quite low (0.311 Å), and the value decreased to 0.183 Å upon removal of the substituents on the nitrogen atoms from the comparison. The computed structure of **10** strongly resembled that of **11** (Figure S17), with negligible variations in bond lengths and angles. This indicated the scarce influence of the different substituents on the NHC ligands.

**Figure 2:**
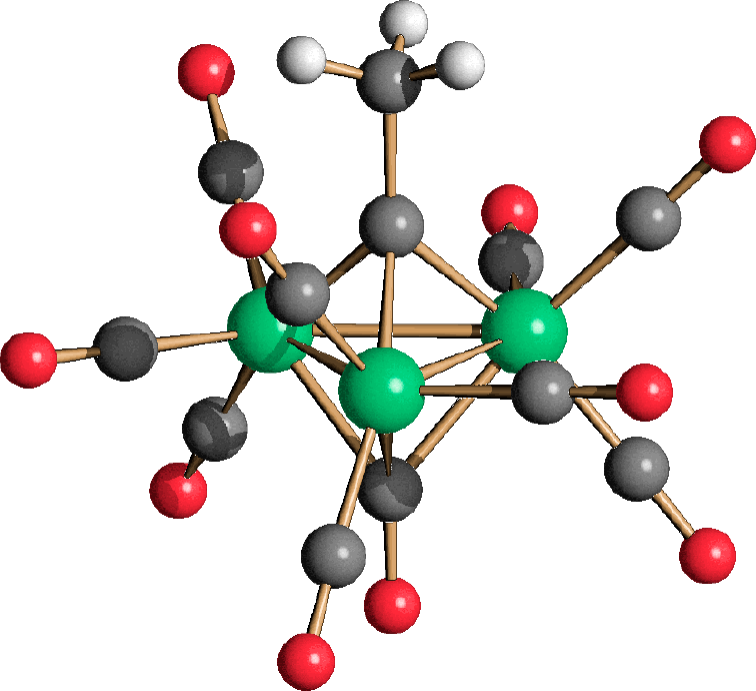
Molecular structure of **12** (green, Fe; red, O; gray, C; white, H). Selected bond lengths (angstroms): Fe–Fe, 2.5285(5)–2.5458(5); Fe–C_ethylidyne_, 1.940(2)–1.960(2); Fe–C(O)_bridging_, 2.015(2)–2.077(2); Fe–C(O)_terminal_, 1.766(3)–1.811(3); C–C_ethylidyne_, 1.497(3).

Besides **11** which was the major product, a few crystals of [NEt_4_][Fe_3_(CO)_10_(CCH_3_)] were isolated as side products of the thermal decomposition of **2** in CH_2_Cl_2_, and their nature was completely revealed by X-ray crystallography. These crystals contained the μ_3_-ethylidyne cluster [Fe_3_(CO)_10_(CCH_3_)]^−^ (**12**) (Figure 2), whose synthesis was previously reported, whereas its structure, to the best of our knowledge, has not been described previously.^33^ The molecular structure of **12** was composed of a triangular Fe_3_ core, bonded to nine terminal CO ligands (three per Fe atom), one μ_3_-ethylidyne, and one μ_3_-CO. The μ_3_-ethylidyne ligand was previously found on related triiron carbonyl clusters, such as Fe_3_(CO)_8_(Cp)(CCH_3_),^34^ H_3_Fe_3_(CO)_9_(CCH_3_),^35^ Fe_3_(CO)_9_(COCH_3_)(CCH_3_),^36^ and Fe_3_(CO)_10_(CuPPh_3_)(CCH_3_),^37^ as well as the tetrairon cluster [Fe_4_(CO)_12_(CCH_3_)]^−^.^38^ It is noteworthy that the closely related Fe_3_(CO)_10_(CuPPh_3_)(CCH_3_),^37^ which formally arose from the addition of a [CuPPh_3_]^+^ fragment to **12**, displayed nine terminal ligands and one edge-bridging μ-CO ligand, instead of a face-bridging μ_3_-CO. A similar stereochemistry of the carbonyl ligands was found in the μ_3_-methylidyne cluster [Fe_3_(CO)_10_(CH)]^−^.^39^ The bonding parameters of **12** (see the legend of Figure 2) were similar to those previously reported for related clusters.^34−38^ The μ_3_-CO [Fe–C(O)_bridging_, 2.015(2)–2.077(2) Å] and μ_3_-CCH_3_ [Fe–C_ethylidyne_, 1.940(2)–1.960(2) Å] ligands were symmetrically bonded to the Fe_3_ triangle, and the C–C_ethylidyne_ distance [1.497(3) Å] was as expected for a single bond.

**12** was previously synthesized from the reaction of [HFe_3_(CO)_11_]^−^ with acetylene.^33^ The mechanism for the formation of **12** as a side product along with the thermal decomposition of **2**, which afforded **11** as the major product, was not clear. It probably involved the oxidation of **2** by means of CH_2_Cl_2_ as described above followed by removal of the AuIPr fragment and rearrangement of the cluster core. Unfortunately, due to the very low yields, it was not possible to further elucidate the mechanism.

### 2.2 Syntheses and Characterization of [Au_3_{Fe(CO)_4_}_2_(PPh_3_)_2_]^−^ (**15**)

**Figure 3:**
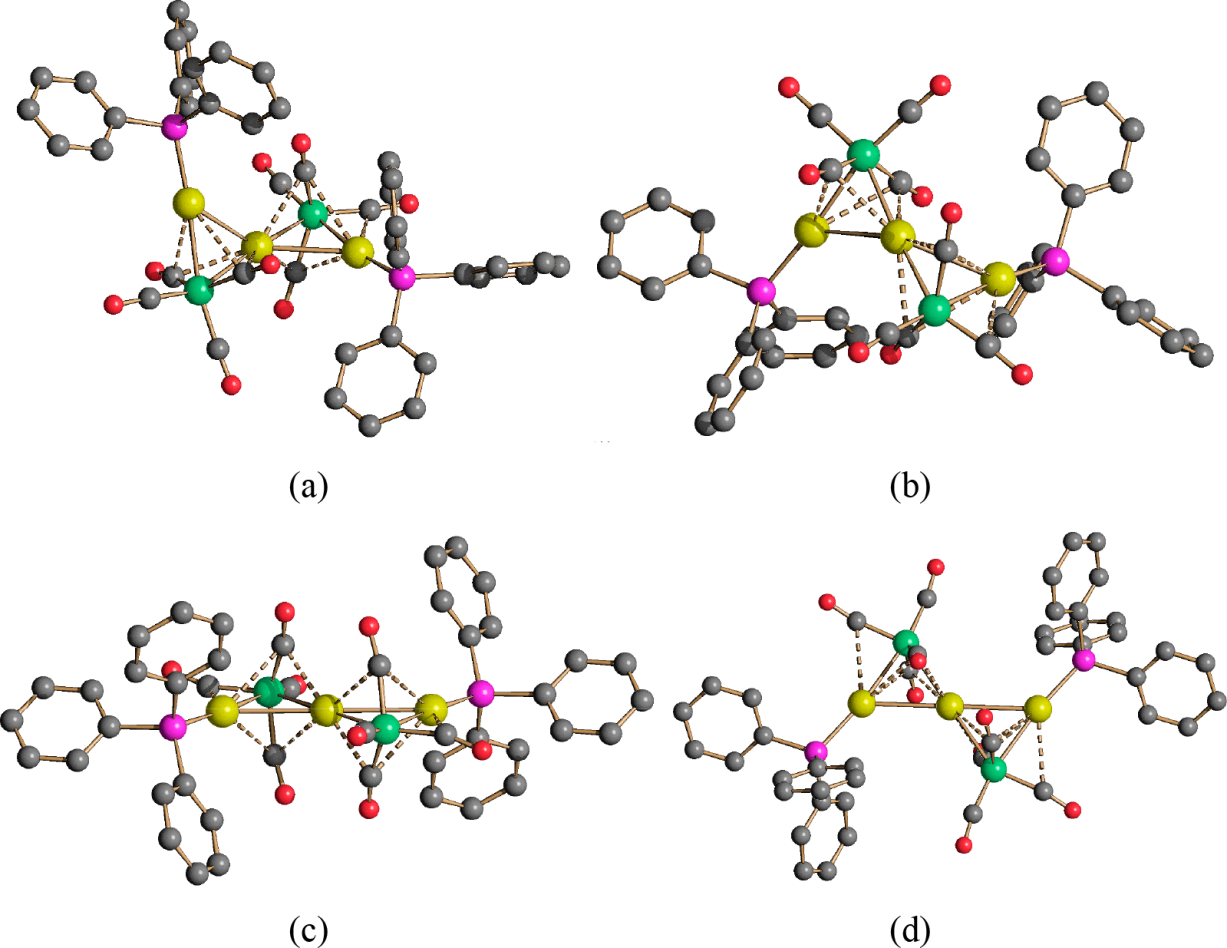
Molecular structures of the two isomers of **15**. The two isomers were present within the crystal in a 2:1 **15a**:**15b** ratio. Two views of isomer **15a** are shown in panels a and b, and two views of isomer **15b** are shown in panels c and d. Au–C(O) contacts [2.34(6)–2.87(8)Å for **15a** and 2.56(3)–2.89(3) Å for **15b**] are represented as dashed lines. Hydrogen atoms have been omitted for the sake of clarity (green, Fe; yellow, Au; purple, P; blue, N; red, O; gray, C).

**Figure 4:**
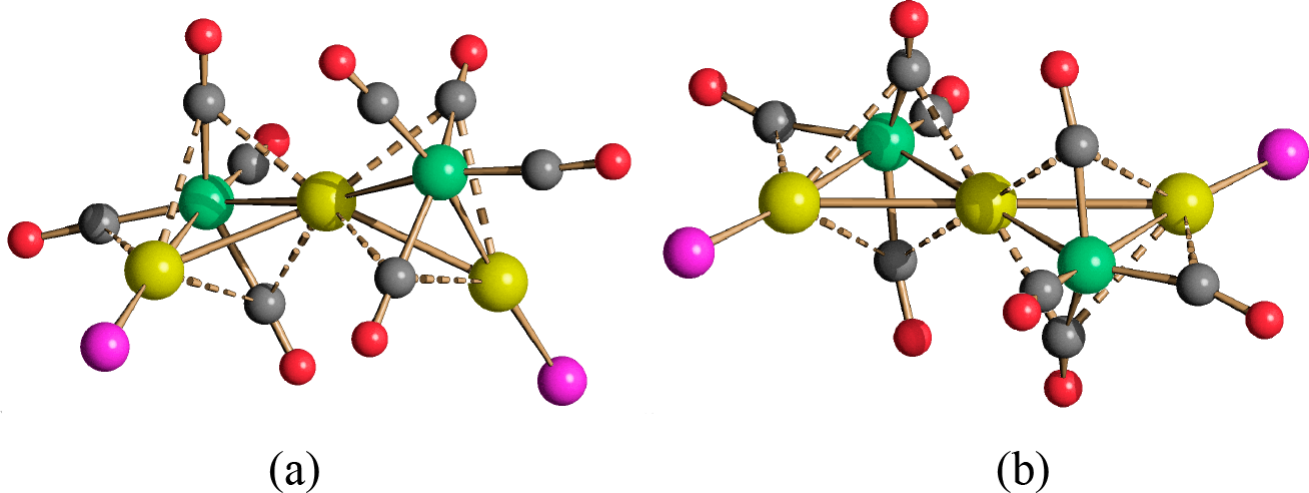
Au_3_Fe_2_(CO)_8_P_2_ cores of (a) **15a** and (b) **15b** (green, Fe; yellow, Au; purple, P; red, O; gray, C).

Complex **6**, which contained mixed IMes/PPh_3_ ligands, was not very stable in polar solvents at room temperature.^4^ Indeed, its ^31^P{^1^H} NMR spectrum in a CD_3_COCD_3_ solution displayed a major resonance (δ_P_) 40.8 ppm attributable to **6**, accompanied by minor resonances at 40.1 and 38.5 ppm (Figures S8 and S9). These resonances corresponded to Fe(CO)_4_(AuPPh_3_)_2_ (**14**) and a new species **15**, respectively. The former was a byproduct of the synthesis of **6** as previously reported,^4^ whereas the formation of **15** arose from partial decomposition (ionization) of **6**. Indeed, after this mixture had been heated in CH_3_CN at 80 °C for 3 h, the intensity of the resonance at 40.8 ppm (δ_P_) considerably decreased, whereas the resonance at 38.5 ppm (δ_P_) became the major one (Figure S9). This indicated an almost complete conversion of **6** into **15**. This new compound was completely characterized by IR, ^1^H, ^13^C{^1^H}, and ^31^P{^1^H} NMR spectroscopy (Figures S10–S12), and its structure was determined by single-crystal X-ray diffraction as its [Au(IMes)_2_][**15**]·0.67CH_2_Cl_2_ salt (Figures 3 and 4 and Table S1). The latter was composed of [Au(IMes)_2_]^+^ cations and [Au_3_{Fe(CO)_4_}_2_(PPh_3_)_2_]^−^ anions (**15**), according to eq 3.
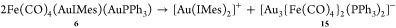
3Within the crystals of [Au(IMes)_2_][**15**]·0.67CH_2_Cl_2_, two isomers of anion **15** were present in a 2:1 ratio (termed isomers **15a** and **15b**, respectively). Both isomers were composed of a Au_3_ core bonded to two μ-Fe(CO)_4_ units and two terminal PPh_3_ ligands. The Au_3_ core of **15a** displayed a V-shaped geometry [∠Au–Au–Au, 132.00(4)°], whereas it adopted a linear arrangement in **15b** with the central Au atom located on an inversion center [∠Au–Au–Au, 180.00(10)°]. The structure of isomer **15b** was similar to that previously reported for **13**.^3^ Both isomers displayed two aurophilic Au···Au contacts [2.9353(13) and 2.8855(14) Å for **15a** and 2.9177(14) and 2.9177(14) Å for **15b**] as well as sub-van der Waals Au···C(O) contacts [2.34(6)–2.87(8) Å for **15a** and 2.56(3)–2.89(3) Å for **15b**].

The presence in the solid state of two isomers of **15** prompted a variable-temperature ^31^P{^1^H} NMR investigation. Unfortunately, a single resonance was observed at all of the temperatures examined (193–298 K), suggesting a fast exchange between **15a** and **15b** in solution.

The structures of **15a** and **15b** were also optimized by DFT calculations. The RMSD values of the computed [Fe_2_Au_3_] cores with respect to the experimental data were 0.129 and 0.064 Å for **15a** and **15b**, respectively. The deviations could mainly be attributed to a slight overestimation of the Au–Au distances, caused by the known weakness of DFT methods in predicting dispersion interactions such as the aurophilic one.^40^ Despite this limit, the computed energy difference between the two isomers was 0.9 kcal mol^–1^, **15a** being slightly more stable than **15b**, in agreement with the observed fast exchange. In both clusters, no (3, −1) bond critical point (bcp) for Au–Au interactions was found, the gradient norm of electron density being greater than zero along the Au–Au bonds (minimum gradient values were 0.005 and 0.004 au for **15a** and **15b**, respectively). This result, which suggested a delocalized dispersion interaction, was in line with the data previously reported for **8**.^7^ The (3, −1) bcp was instead found for the Fe–Au bonds, and relevant data are listed in Table S2 and compared with those obtained for compounds **10** and **11**. All Fe–Au bcp’s were characterized by negative energy density (*E*) values, while the Laplacian of electron density (∇^2^ρ) was positive, in agreement with Bianchi’s definition of M–M bonds.^41^ ρ and *V* values of **15a** and **15b** were closely comparable, and the bonds with terminal Au atoms were stronger than those with the central Au. The data listed in Table S2 indicated that the different mode of binding of Au in **10** and **11** caused a slight decrease in the Fe–Au bond strength. With respect to the charge distribution, the three Au atoms in **15a** and **15b** had very similar Hirshfeld partial charges, in the ranges of 0.060–0.064 au for **15a** and 0.057–0.065 au for **15b**, as expected considering the formal homogeneity of the oxidation states.

### 2.3 Syntheses and Characterization of [Au_16_S{Fe(CO)_4_}_4_(IPr)_4_]^*n*+^ (**17**)

**4** was recovered almost intact also after heating in dmso at 140 °C for 5 h. It started to show a partial decomposition only after prolonged heating above 150 °C in dmso. Among the decomposition products, it was possible to isolate a few crystals of [NEt_4_]_2_[Fe_3_S(CO)_9_] ([NEt_4_][**9**]), [Au(IPr)_2_][HFe(CO)_4_] ([Au(IPr)_2_][**16**]), and [Au_16_S{Fe(CO)_4_}_4_(IPr)_4_][BF_4_]_n_·solvent ([**17**][BF_4_]_*n*_·solvent). The presence of [BF_4_]^−^ anions in the latter salt was due to the use of [NEt_4_][BF_4_] during workup of the reaction mixture.

Anions **9** and **16** were previously reported, and^26,42^ therefore, their structures will not be discussed further. Their crystal data were deposited within the Cambridge Crystallographic Data Centre, and a representation of the molecular structure of **9** is included as Figure S16.

**Figure 5:**
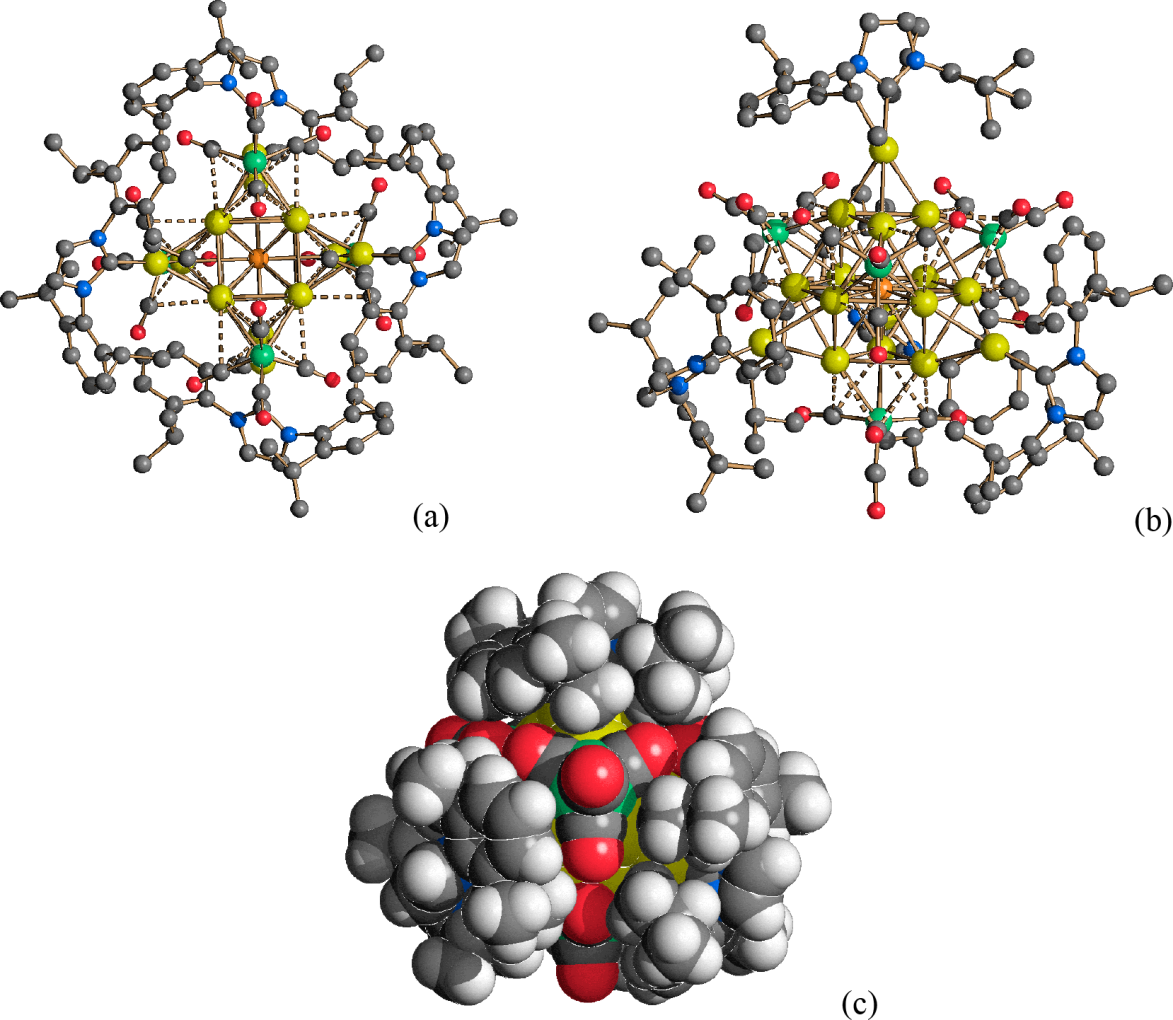
Molecular structure of **17**: (a and b) two different views as well as (c) the space filling model. Au–C(O) contacts [2.636(4)–2.723(4) Å] are represented as dashed lines. Hydrogen atoms have been included only in the space filling model (green, Fe; yellow, Au; orange, S; blue, N; red, O; gray, C; white, H).

Formation of **9** was rather interesting because it suggested that S atoms were somehow generated from dmso after the prolonged thermal treatment of **4**. This was in keeping with the formation of the new species **17**, which contained an interstitial sulfur atom. Compound **17** was formed in only trace amounts, and because of this, only very few small crystals were grown. This allowed the complete determination of the molecular structure of the cluster molecule (Figure 5 and Table S3), which occupied 78% of the unit cell volume. The remaining 22% of the volume of the unit cell was likely to be occupied by cations/anions and/or solvent molecules (Figure S17), whose nature was not determined.

Despite the fact that **17** was obtained in low yields, it was also possible to characterize it by multinuclear NMR techniques. ^1^H and ^13^C{^1^H} NMR analyses were in agreement with the presence of CO and IPr ligands (Figures S13 and S14). More interestingly, the ^19^F NMR spectrum of **17** (Figure S15) displayed the typical resonance of the [BF_4_]^−^ anion. Therefore, **17** was better formulated as a cationic species, and because of this, its crystals were denoted [**17**][BF_4_]_*n*_·solvent.

**Figure 6:**
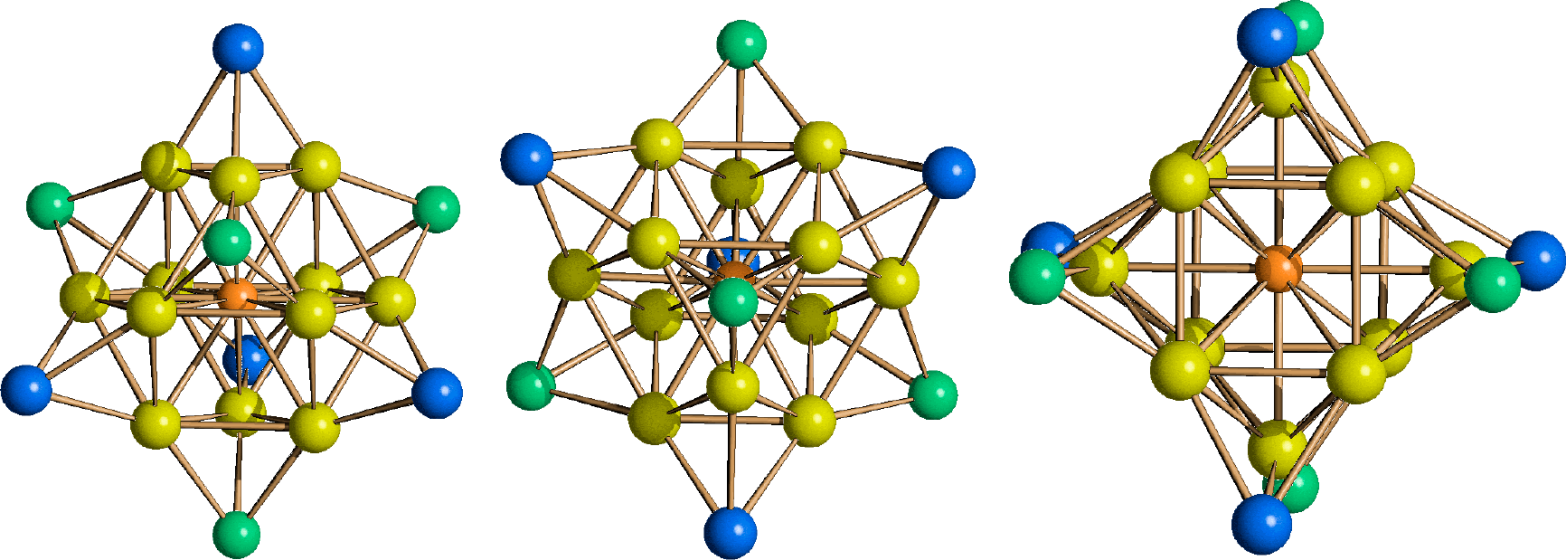
Three different views of the Au_16_S core of **17** (green, Fe; yellow, Au atoms of the Au_12_ cubeoctahedron; blue, Au atoms of the μ_3_-AuIPr fragments; orange, S).

**17** consisted of a Au_12_-cubeoctahedron centered by a μ_12_-S atom, whose surface was decorated with four μ_3_-Fe(CO)_4_ and four μ_3_-AuIPr fragments with a pseudo-*T*_*d*_ symmetry (Figure 6). A related structure, where a μ_12_-S atom was encapsulated within a Cu_12_-cubeoctahedral cage, was recently reported for the neutral [Cu_12_(μ_12_-S)(S_2_CN^n^Bu_2_)_6_(C≡CPh)_4_] cluster.^43^ As in the case of [Cu_12_(μ_12_-S)(S_2_CN^n^Bu_2_)_6_(C≡CPh)_4_], the Au–S distances [2.7641(13)–2.7995(16) Å, average of 2.777(3) Å] of **17** were rather elongated in light of the high coordination number of the interstitial μ_12_-S atom. For comparison, the sums of the covalent and van der Waals radii of Au and S were 2.38 and 3.46 Å, respectively.^44^ Prior of the isolation of **17**, the highest coordination number observed for S with Au was four, and the corresponding Au–S distances were considerably shorter (2.30–2.42 Å).^45,46^

The tangential Au–Au contacts [2.702(2)–2.874(2) Å, average of 2.753(6) Å] were more dispersed compared to the more localized Au–Au contacts involving the μ_3_-AuIPr fragments [2.724(2)–2.733(2) Å, average of 2.728(3) Å]. Similarly, the Au–Fe distances [2.625(5)–2.650(5) Å, average of 2.636(9) Å] displayed by **17** that presented μ_3_-Fe(CO)_4_ groups were significantly longer than those found in clusters containing μ_2_-Fe(CO)_4_ fragments such as **15** [2.529(3)–2.601(11) Å, average of 2.564(8) Å].

Molecular gold nanoclusters stabilized by ligands have been extensively studied in recent years.^11−22^ Au_13_ and Au_12_M cages often adopted icosahedral structures, and a few clusters displaying a cubeoctahedral structure were reported.^15^ This point was also computationally investigated, showing that, depending on the central atom, Au_12_M clusters could adopt *I*_*h*_ (icosahedron) or *O*_*h*_ (cubeoctahedron) symmetry.^47^

DFT calculations were carried out on models of compound **17**. The substituents on the nitrogen atoms of the NHC ligands were replaced by methyl groups to reduce the computational effort. The coordinates of the other atoms were obtained from X-ray data. The singlet multiplicity was always maintained, and the charge was varied from 2+ to 6+. The most stable electronic structure resulted in the most reduced one, that is 2+. The 4+ and 6+ cations were less stable by 0.9 and 2.2 au, respectively. For this reason, the formula [Au_16_S{Fe(CO)_4_}_4_(IPr)_4_]^2+^ was proposed. The computed energy gap between frontier orbitals in the model compound was quite high, 3.9 eV.

**Figure 7:**
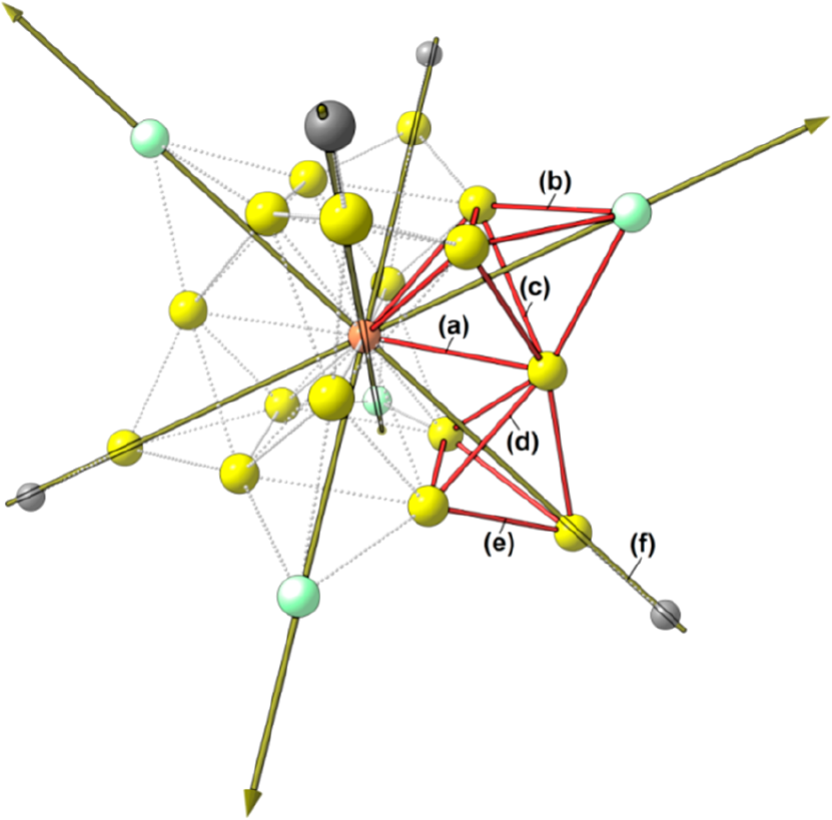
Structure of **17** with one [FeAu_3_] and one [Au_4_] tetrahedron highlighted. The CO ligands have been removed for the sake of clarity. Only the donor atoms of the NHC ligands are depicted. The four C_3_ axes are shown. Different types of bonds involving the Au centers are labeled. Color map: Au, yellow; S, orange; Fe, green; C, gray.

The approximate *T*_*d*_ symmetry was confirmed by all of the population analyses, and the four C_3_ axes are reported in Figure 7 for the sake of clarity. The compound can be considered to be composed of four [FeAu_3_] tetrahedra, each one forming three bonds with the central sulfur. One of the [FeAu_3_] tetrahedra and its bonds with S are colored red in Figure 7. The [FeAu_3_] tetrahedra were interconnected by Au–Au bonds, and each [AuNHC] fragment (NHC = 1,3-dimethylimidazol-2-ylidene) was bonded to three Au atoms belonging to different [FeAu_3_] tetrahedra, with the formation of [Au_4_] tetrahedra, one of them highlighted in Figure 7. The bonds involving the Au centers can therefore be grouped into six types, as depicted in Figure 7: (a) Au–S, (b) Au–Fe, (c) Au–Au in [FeAu_3_], (d) Au–Au in [Au_4_], between iron-bonded centers, (e) Au–Au in [Au_4_] involving the [AuNHC] fragment, and (f) Au-NHC. Average values concerning the (3, −1) bcp are listed in Table S4. It is worth noting that the AIM analysis was unable to find the (3, −1) bcp associated with Au–CO interactions.

As for the previously discussed compounds, all of the bcp’s considered in Table S4 were characterized by negative *E* and positive ∇^2^ρ values, in agreement with the definition of M–M and dative bonds.^41^ The Au–Au bonds in the [Au_4_] tetrahedra had similar ρ and *V* values at bcp, thus indicating comparable strength. The Au–Au interactions in the [FeAu_3_] fragments were comparatively slightly weaker. Considering the Au–S, Au–Fe, and Au–Au bonds, the *V* values fell in a quite limited range, between −0.034 and −0.062 au, while the average *V* value related to the Au-NHC bcp was meaningfully more negative, −0.193 au. The picture coming from the AIM analysis was that the Au, S, and Fe atoms in **17** formed a network of bonds having roughly comparable strength.

The partial charges on the Au atoms obtained from the Hirshfeld population analysis were between 0.059 and 0.126 au. The less positive values were related to the NHC-bonded Au atoms, probably because of the donation from the ligands. The maximum charge variation among the other Au centers was 0.03 au, supporting a homogeneous distribution of electron density. Quite interestingly, also the Hirshfeld charge on sulfur was slightly positive (0.045 au). Therefore, AIM and Hirshfeld data suggested that the behavior of the central sulfur was roughly comparable to that of the surrounding Au atoms. Finally, as expected, the Hirshfeld charge on Fe atoms was negative, −0.176 au.

The electron count of **17** was based on the following assumptions. The μ_3_-AuIPr fragments were considered to contribute one electron each, being isolobal to μ_3_-H. The μ_3_-Fe(CO)_4_ groups were usually described in the literature as four-electron donors.^48^ The interstitial μ_6_-S atom was considered to contribute with all of its six valence electrons. Therefore, if **17** was a dication, as inferred from DFT calculations, it should possess 156 [11 × 12 (Au) + 6 × 1 (μ_6_-S) + 4 × 1 (μ_3_-AuIPr) + 4 × 4 (μ_3_-Fe(CO)_4_) – 2 (charge +2)] cluster valence electrons (CVEs). The expected CVEs depended of the model adopted. According to the EAN (effective atomic number) rule, a cubeoctahedron should have 168 CVE. PSEPT (polyhedral skeletal electron pair theory) predicted 170 CVE by interpreting a cubeoctahedron as a four-connected polyhedron. Conversely, assuming that radial bonding predominates, on the basis of Mingos rules a cubeoctahedron should have 162 CVE.^48,49^ In this respect, **17** appeared to be electron poor, as often happened for gold clusters.^49^

## 3 Conclusions

Low-nuclearity Fe–Au compounds **1–7** thermally decomposed to high-nuclearity species. The obtained Fe–Au products could be grouped within the following categories. (1) Products **8**, **13**, and **15** were the result of ionization and rearrangement of the starting species. Thus, they retained the original oxidation states of the metals, that is, Au(+1) and Fe(−2). (2) **10** and **11** resulted from oxidation of iron from −2 to −1, whereas gold retained the original +1 oxidation state. (3) The unique species **17** (even if obtained in very low yields) formally contained Fe(−2), whereas the oxidation state of Au was between 0 and +1. This assignment was based on the assumption that, as usually found in Au–Fe carbonyl clusters,^1,3−8^ the Fe(CO)_4_ fragments retained their original dianionic nature.

All of the heterometallic clusters reported contained strong Fe–CO, Fe–Au, Au–P, and Au–NHC bonds as well as weak Au···Au interactions. AIM analyses and DFT studies pointed out that the Au···Au interactions in such heterometallic clusters were mainly dispersion-driven. In addition, the different behavior of IMes and IPr derivatives was essentially due to steric effects, because no appreciable electronic difference is evidenced by population analyses based on DFT calculations, as previously reported.^4^

IPr-containing species were in general more stable than IMes-containing species. In all cases, even when **1–7** were heated to 160 °C, the formation of carbido clusters was not observed. This was probably due to the presence of the AuNHC fragments, because in their absence, anions of iron carbonyls afforded Fe_5_ and Fe_6_ carbido clusters.^50,51^

## 4 Experimental Section

### 4.1 General Experimental Procedures

All reactions and sample manipulations were carried out using standard Schlenk techniques under nitrogen and in dried solvents. All of the reagents were commercial products (Aldrich) of the highest available purity and used as received, except **1–7**, which were prepared according to the literature.^3,4^ Analyses of C, H, and N were obtained with a Thermo Quest Flash EA 1112NC instrument. IR spectra were recorded on a PerkinElmer Spectrum One interferometer in CaF_2_ cells. Structure drawings were performed with SCHAKAL99.^52^

### 4.2 Thermal Decomposition of [NEt_4_][Fe(CO)_4_(AuNHC)] [NHC = IMes (**1**) and IPr (**2**)] in Nonchlorinated Solvents

A solution of [NEt_4_][**1**] (0.530 g, 0.663 mmol) in dmso (10 mL) was heated at 130 °C for 3 h, and the reaction monitored by IR spectroscopy. Then, a saturated solution of [NEt_4_]Br in H_2_O (40 mL) was added to complete precipitation. The resulting solid was recovered by filtration, washed with H_2_O (3 × 15 mL) and toluene (3 × 15 mL), and extracted with acetone (15 mL). A microcrystalline powder of [NEt_4_]_3_[**8**] was obtained after removal of the solvent under reduced pressure (0.134 g yield, 41% based on Fe, 41% based on Au). The compound was identified by comparison of its IR data with those reported in the literature.^7^

Decomposition of [NEt_4_][**2**] to produce **8** occurred at 150 °C in dmso. With a further increase in the temperature to 160–170 °C, a complex mixture of decomposition products was formed, among which **9** was the major species detected by IR spectroscopy.

### 4.3 Synthesis of [Fe_2_(CO)_8_(AuNHC)]^−^ [NHC = IMes (**10**) or IPr (**11**)]

A solution of [NEt_4_][**2**] (0.530 g, 0.600 mmol) in CH_2_Cl_2_ (20 mL) was heated at 40 °C for 4 h, and the reaction monitored by IR spectroscopy. Then, the solvent was removed under reduced pressure, and the residue washed with H_2_O (3 × 15 mL) and extracted with toluene (10 mL). Crystals of [NEt_4_][**11**]·1.5toluene suitable for X-ray crystallography were obtained by slow diffusion of *n*-pentane (25 mL) on the toluene solution (0.207 g yield, 58% based on Fe, 29% based on Au).

A few crystals of [NEt_4_][**12**] were isolated as side products of the thermal decomposition of **2** in CH_2_Cl_2_, and their nature was completely revealed by X-ray crystallography.

[NEt_4_][**11**]·1.5toluene. C_53.5_H_68_AuFe_2_N_3_O_8_ (1189.77). Calcd (%): C, 59.98; H, 5.76; N, 3.53. Found: C, 60.12; H, 5.38; N, 3.21. IR (nujol, 293 K): ν_CO_ 2004(w), 1956(s), 1923(ms), 1895(vs), 1880(sh) cm^–1^. IR (dmso, 293 K): ν_CO_ 2004(w), 1958(s), 1913(sh), 1900(vs), 1728(ms) cm^–1^. IR (CH_3_CN, 293 K): ν_CO_ 2006(w), 1960(s), 1903(vs), 1727(ms) cm^–1^. IR (acetone, 293 K): ν_CO_ 2004(w), 1958(s), 1912(sh), 1902(vs) cm^–1^. IR (toluene, 293 K): ν_CO_ 2005(w), 1963(s), 1900(vs), 1721(m) cm^–1^. IR (CH_2_Cl_2_, 293 K): ν_CO_ 2007(w), 1961(s), 1905(vs), 1715(ms) cm^–1^. IR (thf, 293 K): ν_CO_ 2002(w), 1959(s), 1904(vs), 1720(m) cm^–1^. ^1^H NMR (CD_3_COCD_3_, 298 K): δ 7.50–7.24 (m, 8H, CH_Ar_ + CH_imid_), 3.44 (q, ^2^*J*_HH_ = 6.2 Hz, 8H, NC*H*_*2*_CH_3_), 2.95 [sept, ^2^*J*_HH_ = 6.8 Hz, 4H, C*H*(CH_3_)_2_], 1.36 [d, ^2^*J*_HH_ = 6.8 Hz, 12H, CH(C*H*_3_)_2_], 1.35 (t, ^2^*J*_HH_ = 6.2 Hz, 12H, NCH_2_C*H*_*3*_), 1.15 [d, ^2^*J*_HH_ = 6.8 Hz, 12H, CH(C*H*_3_)_2_]. ^13^C{^1^H} NMR (CD_3_COCD_3_, 298 K): δ 231.5 (CO), 200.4 (C–Au), 145.4, 135.9, 129.4, 123.7, 123.3 (C_Ar_ and CH_imid_), 51.9 (N*C*H_2_CH_3_), 28.2 [*C*H(CH_3_)_2_], 23.9, 23.3 [CH(*C*H_3_)_2_], 6.7 (NCH_2_*C*H_3_).

The thermal decomposition of **1** under the same experimental conditions described above afforded [Fe_2_(CO)_8_(AuIMes)]^−^ (**10**). IR (CH_2_Cl_2_, 293 K): ν_CO_ 2000(w), 1959(s), 1899(vs), 1712(ms) cm^–1^.

### 4.4 Synthesis of [NBu_4_][Au_3_Fe_2_(CO)_8_(IMes)_2_]·CH_3_COCH_3_ ([NBu_4_][**13**]·CH_3_COCH_3_)

A large excess of [NBu_4_][BF_4_] was added as a solid to a solution of **3** (0.190 g, 0.531 mmol) in dmf (20 mL), and the mixture stirred at 100 °C for 1 h. Then, the orange solution was cooled to room temperature, and H_2_O (60 mL) was added until complete precipitation occurred. The solid was recovered by filtration, washed with H_2_O (40 mL), and extracted in acetone (10 mL). Needle-like pale yellow crystals of [NBu_4_][**13**]·CH_3_COCH_3_ suitable for X-ray analyses were obtained by slow diffusion of *n*-hexane (30 mL) on the acetone solution (122 g yield, 25% based on Fe).^3^

C_69_H_90_Au_3_Fe_2_N_5_O_9_ (1836.06). Calcd (%): C, 45.11; H, 4.94; N, 3.81; Fe, 6.09; Au, 32.19. Found: C, 45.41; H, 5.12; N, 3.62; Fe, 6.31; Au, 31.85. IR (nujol, 293 K): ν_CO_ 1948(vs), 1877(sh), 1867(s), 1836(sh), 1712(m) cm^–1^. IR (acetone, 293 K): ν_CO_ 1968(sh), 1947(m), 1924(m), 1872(s) cm^–1^. ^1^H NMR (CD_2_Cl_2_, 298 K): δ 7.12 (s, 8H, CH_imid_), 6.94 (s, 16H, CH_Ar_), 3.20 (br, 8H, NC*H*_*2*_CH_2_CH_2_CH_3_), 2.47 (s, 24H, CH_3_), 1.74 (s, 48H, CH_3_), 1.65 (br, 8H, NCH_2_*CH*_*2*_CH_2_CH_3_), 1.47 (br, 8H, NCH_2_CH_2_*CH*_*2*_CH_3_), 1.01 (br, 12H, NCH_2_CH_2_CH_2_*CH*_*3*_). ^13^C{^1^H} NMR (CD_2_Cl_2_, 298 K): δ 220.9 (CO), 185.3 (C–Au), 139.4, 134.6, 134.1, 129.0 (C_Ar_), 122.8 (CH_imid_), 58.6 (N*C*H_2_CH_2_CH_2_CH_3_), 23.7 (NCH_2_*C*H_2_CH_2_CH_3_), 19.6 (NCH_2_CH_2_*C*H_2_CH_3_), 20.9, 16.9 (CH_3_), 13.3 (NCH_2_CH_2_CH_2_*C*H_3_).

### 4.5 Synthesis of [NMe_4_]_2_[Au(IMes)_2_][Au_3_{Fe(CO)_4_}_3_] ([NMe_4_]_2_[Au(IMes)_2_][**8**])

A solution of **3** (0.450 g, 0.384 mmol) in dmso (15 mL) was heated at 130 °C for 0.5 h, and the reaction monitored by IR spectroscopy. Then, a saturated solution of [NMe_4_]Cl in H_2_O (40 mL) was added to complete precipitation. The resulting solid was recovered by filtration, washed with H_2_O (3 × 15 mL) and toluene (3 × 15 mL), and extracted with acetone (15 mL). Crystals of [NMe_4_]_2_[Au(IMes)_2_][**8**] suitable for X-ray crystallography were obtained by slow diffusion of *n*-hexane (35 mL) on the acetone solution (0.14 g yield, 52% based on Fe, 36% based on Au).^7^

[NEt_4_]_2_[Au(IMes)_2_][**8**]·CH_3_COCH_3_ was obtained following a similar procedure and employing [NEt_4_]Br instead of [NMe_4_]Cl.

[NMe_4_]_2_[Au(IMes)_2_][**8**]. C_62_H_72_Au_4_Fe_3_N_6_O_12_ (2048.67). Calcd (%): C, 36.32; H, 3.54; N, 4.10. Found: C, 36.14; H, 3.71; N, 3.89. IR (nujol, 293 K): ν_CO_ 1970(m), 1932(s), 1843(s) cm^–1^. IR (dmso, 293 K): ν_CO_ 1974(w), 1930(s), 1879(s) cm^–1^. IR (CH_2_Cl_2_, 293 K): ν_CO_ 1975(w), 1929(s), 1877(s) cm^–1^. IR (CH_3_CN, 293 K): ν_CO_ 1929(s), 1867(s) cm^–1^. IR (acetone, 293 K): ν_CO_ 1969(w), 1928(s), 1864(s) cm^–1^. ^1^H NMR (CD_3_CN, 298 K): δ 7.25 (s, 4H, CH_imid_), 6.98 (s, 8H, CH_Ar_), 3.17 (s, 24H, NMe_4_), 2.45 (s, 12H, CH_3_), 1.72 (s, 24H, CH_3_). ^13^C{^1^H} NMR (CD_2_Cl_2_, 298 K): δ 224.4 (CO), 185.3 (C–Au), 139.7, 135.0, 134.6, 129.2 (C_Ar_), 123.4 (CH_imid_), 55.6 (^1^*J*_CN_ = 3.9 Hz, NMe_4_), 20.6, 16.7 (CH_3_).

### 4.6 Synthesis of [Au(IMes)_2_][Au_3_{Fe(CO)_4_}_2_(PPh_3_)_2_]·0.67CH_2_Cl_2_ ([Au(IMes)_2_][**15**]·0.67CH_2_Cl_2_)

A solution of **6** (0.220 g, 0.188 mmol) in CH_3_CN (15 mL) was heated at 80 °C for 3 h, and the reaction monitored by IR spectroscopy. Then, a saturated solution of [NEt_4_]Br in H_2_O (40 mL) was added to complete precipitation. The resulting solid was recovered by filtration, washed with H_2_O (3 × 15 mL) and toluene (3 × 15 mL), and extraced with CH_2_Cl_2_ (15 mL). Crystals of [Au(IMes)_2_][**15**]·0.67CH_2_Cl_2_ suitable fon X-ray crystallography were obtained by slow diffusion of *n*-pentane (35 mL) on the CH_2_Cl_2_ solution (0.110 g yield, 51% based on Fe, 51% based on Au).

C_86.67_H_79.33_Au_4_Cl_1.33_Fe_2_N_4_O_8_P_2_ (2313.64). Calcd (%): C, 44.98; H, 3.46; N, 2.42. Found: C, 45.12; H, 3.71; N, 2.14. IR (nujol, 293 K): ν_CO_ 1977(w), 1953(s), 1887(s), 1864(sh), 1843(sh) cm^–1^. IR (CH_3_CN, 293 K): ν_CO_ 1989(w), 1965(m), 1891(s) cm^–1^. IR (acetone, 293 K): ν_CO_ 1988(w), 1963(m), 1891(s) cm^–1^. ^1^H NMR (CD_3_COCD_3_, 298 K): δ 7.85–6.98 (m, 42 H, CH_Ar_ + CH_imid_ + Ph), 2.46 (s, 12H, CH_3_), 1.76 (s, 24H, CH_3_). ^13^C{^1^H} NMR (CD_3_COCD_3_, 298 K): δ 220.8 (CO), 185.1 (C–Au), 139.3, 134.6, 134.4, 134.2, 130.6, 129.0, 128.9, 128.8, 123.3 (CH_Ar_ + CH_imid_ + Ph), 20.3, 16.4 (CH_3_). ^31^P{^1^H} NMR (CD_3_COCD_3_, 298 K): δ 38.5.

### 4.7 Thermal Decomposition of Fe(CO)_4_(AuIPr)_2_ (4)

**4** was very stable in solution even after being heated at 130–150 °C in dmso. The reactions were periodically monitored by IR spectroscopy, and even after 12–24 h, the main ν_CO_ bands present in the spectra were those attributable to the starting **4**. Then, a saturated solution of [NEt_4_][BF_4_] in H_2_O (40 mL) was added to complete precipitation. The resulting solid was recovered by filtration, washed with H_2_O (3 × 15 mL) and toluene (3 × 15 mL), and extracted with solvents of increasing polarity: CH_2_Cl_2_ (15 mL), thf (15 mL), acetone (15 mL), CH_3_CN (15 mL), and dmso (15 mL). **4** was the main product recovered independent of the experimental conditions. Nonetheless, several attempts at crystallization were made by layering suitable solvents on the solutions mentioned above. Besides the crystals of **4**, these attempts resulted in a few crystals of [Au(IPr)_2_][**16**], [NEt_4_]_2_[**9**], and [**17**][BF_4_]_*n*_·solvent. These were likely to arise from partial decomposition of **4**, which also involved dmso activation and formation of sulfide ions. The crystals of [Au(IPr)_2_][**16**], [NEt_4_]_2_[**9**], and [**17**][BF_4_]_*n*_·solvent were separated from the reaction mixtures and analyzed by X-ray crystallography, as well as IR spectroscopy ([NEt_4_]_2_[**9**] and [**17**][BF_4_]_*n*_·solvent) and ^1^H, ^19^F, and ^13^C{^1^H} NMR spectroscopy ([**17**][BF_4_]_*n*_·solvent).

[NEt_4_]_2_[**9**]. IR (nujol, 293 K): ν_CO_ 1999(m), 1820(s), 1892(s), 1865(m) cm^–1^. IR (CH_3_CN, 293 K): ν_CO_ 1988(m), 1932(s), 1904(m), 1873(w) cm^–1^.

[**17**][BF_4_]_*n*_·solvent. IR (nujol, 293 K): ν_CO_ 1975(s), 1903(m), 1856(w) cm^–1^. IR (CH_2_Cl_2_, 293 K): ν_CO_ 2039(m), 1974(s), 1883(s), 1863(m) cm^–1^. IR (thf, 293 K): ν_CO_ 2037(m), 1975(s), 1899(s), 1885(s), 1867(m) cm^–1^. IR (acetone, 293 K): ν_CO_ 2037(m), 1973(s), 1885(s), 1869(m) cm^–1^. ^1^H NMR (CD_3_COCD_3_, 298 K): δ 7.51 (s, 8H, CH_imid_), 7.43 (t, ^2^*J*_HH_ = 7.7 Hz, 8H, CH_Ar_), 7.26 (d, ^2^*J*_HH_ = 7.7 Hz, 16H, CH_Ar_), 2.67 [sept, ^2^*J*_HH_ = 7.4 Hz, 16H, C*H*(CH_3_)_2_], 1.25 [d, ^2^*J*_HH_ = 7.4 Hz, 48H, CH(C*H*_3_)_2_], 1.17 [d, ^2^*J*_HH_ = 7.4 Hz, 48H, CH(C*H*_3_)_2_]. ^13^C{^1^H} NMR (CD_3_COCD_3_, 298 K): δ 222.3 (CO), 199.2 (C–Au), 150.6, 140.2, 135.0, 128.9, 128.1 (C_Ar_ and CH_imid_), 33.7 [*C*H(CH_3_)_2_], 28.9, 28.7 [CH(*C*H_3_)_2_]. ^19^F NMR (CD_3_COCD_3_, 298 K): δ −151.76, −151.81 ([BF_4_]^−^).

### 4.8 Thermal Decomposition of Fe(CO)_4_(AuIMes)(AuIPr) (**5**)

A solution of **5** (0.450 g, 0.359 mmol) in dmso (15 mL) was heated at 130 °C and the reaction monitored by IR spectroscopy. After 3 h, the IR spectrum showed the typical ν_CO_ absorptions of **8** and the reaction was stopped without any further workup.

### 4.9 Thermal decomposition of Fe(CO)_4_(AuIPr)(AuPPh_3_) (**7**)

A solution of **7** (0.450 g, 0.371 mmol) in dmso (15 mL) was heated at 130 °C, and the reaction monitored by IR spectroscopy. After 5 h, the IR spectrum showed the typical ν_CO_ absorptions of the starting compound **7**. The temperature was increased to 150 °C without any clear evidence of decomposition.

### 4.10 X-ray Crystallographic Study

Crystal data and collection details for [Au(IMes)_2_][**15**]·0.67CH_2_Cl_2_, [Au(IPr)_2_][**16**], [NEt_4_]_2_[**9**], [NEt_4_][**12**], [**17**][BF_4_]_*n*_·solvent, and [NEt_4_][**11**]·1.5toluene are reported in Table S5. The diffraction experiments were carried out on a Bruker APEX II diffractometer equipped with a CCD ([**17**][BF_4_]_*n*_·solvent) or a PHOTON100 ([Au(IMes)_2_][**15**]·0.67CH_2_Cl_2_, [Au(IPr)_2_][**16**], [NEt_4_]_2_[**9**], [NEt_4_][**12**], and [NEt_4_][**11**]·1.5toluene) detector using Mo Kα radiation. Data were corrected for Lorentz polarization and absorption effects (empirical absorption correction SADABS).^53^ Structures were determined by direct methods and refined by full-matrix least squares based on all data using *F*^2^.^54^ Hydrogen atoms were fixed at calculated positions and refined by a riding model. All non-hydrogen atoms were refined with anisotropic displacement parameters, unless otherwise stated.

#### 4.10.1 [Au(IMes)_2_][**15**]·0.67CH_2_Cl_2_

The asymmetric unit of the unit cell contains one cluster anion located on a general position, half of a cluster anion located on an inversion center, one [Au(IMes)_2_]^+^ cation located on a general position, half of a [Au(IMes)_2_]^+^ cation located on an inversion center, and one CH_2_Cl_2_ molecule located at a general position. The CO ligands of the cluster anion located on an inversion center as well as the IMes ligands of the [Au(IMes)_2_]^+^ cation located on an inversion center are disordered. Thus, they have been split into two positions and refined using one occupancy factor per disordered group. The disordered CO ligands and the CH_2_Cl_2_ molecule have been refined isotropically. All C, N, and O atoms have been restrained to have similar *U* parameters (SIMU line in SHELXL, s.u. 0.01) and to isotropic behavior (ISOR line in SHELXL, s.u. 0.01). All of the aromatic C atoms have been constrained to fit regular hexagons (AFIX 66 line in SHELXL). Mainly because of the disorder issues mentioned above, the refined *R*_1_ factor was 0.1276.

#### 4.10.2 [Au(IPr)_2_][**16**]

The asymmetric unit of the unit cell contains half of a [HFe(CO)_4_]^−^ anion and half of a [Au(IPr)_2_]^+^ cation both located on the 2-fold axis. The N and C atoms of the IPr ligands have been restrained to have similar *U* parameters (SIMU line in SHELXL, s.u. 0.02). The [HFe(CO)_4_]^−^ anion is disordered over two equally populated and symmetry-related positions. Because of this disorder, it has not been possible to locate the hydride ligand.

#### 4.10.3 [NEt_4_]_2_[**9**]

The asymmetric unit of the unit cell contains two cluster anions and four [NEt_4_]^+^ cations located on general positions. The crystals are racemically twinned with a refined batch factor of 0.32(2). Two [NEt_4_]^+^ cations are disordered, and thus, they have been split into two positions each and refined anisotropically with one occupancy factor per disordered unit. The disordered cations have been restrained to have similar *U* parameters (SIMU line in SHELXL, s.u. 0.01), similar geometries (SAME line in SHELXL, s.u. 0.02), and isotropic behavior (ISOR line in SHELXL, s.u. 0.01).

#### 4.10.4 [NEt_4_][**12**]

The asymmetric unit of the unit cell contains one cluster anion and one [NEt_4_]^+^ cation both located on general positions.

#### 4.10.5 [**17**][BF_4_]_*n*_·Solvent

The asymmetric unit of the unit cell contains one-fourth of a cluster molecule located on 4. A total potential solvent accessible void of 1794 Å^3^ (∼22% of the cell volume) remains within the unit cell after refinement. These voids are organized in infinite channels parallel to the crystallographic *c* axis. In view of the fact that the crystals are very small, even if the data have been collected at 100 K with 120 s per frame, it has not been possible to crystallographically identify any molecule within these channels. For the same reasons, the final *R*_1_ factor was 0.1799. It must be remarked that, even if hundreds of Fourier peaks are included during refinement (PLAN 200 or even higher in SHELXL), all of them are located close to the cluster molecule and not within the void channels. In addition, ^1^H and ^13^C NMR analyses of the crystals dissolved in *d*_6_-acetone did not show any significant peaks apart those attributable to the cluster molecule. Conversely, ^19^F NMR analyses clearly pointed out the presence of [BF_4_]^−^ anions. Nonetheless, because it was not possible to locate and refine such anions within the crystal structure, these voids were treated using the SQUEEZE routine of PLATON.^55^ All phenyl rings have been constrained to fit regular hexagons (AFIX 66 line in SHELXL).

#### 4.10.6 [NEt_4_][**11**]·1.5Toluene

The asymmetric unit of the unit cell contains one cluster anion located at a general position, one [NEt_4_]^+^ cation located at a general position, one toluene molecule located at a general position, and one toluene molecule located at an inversion center disordered over two symmetry-related positions (occupancy factor of 0.5). All of the C, O, and N atoms have been restrained to have similar *U* parameters (SIMU line in SHELXL, s.u. 0.01). The C–N and C–C distances of the [NEt_4_]^+^ cation have been restrained to be similar (SADI line in SHELXL, s.u. 0.02). The aromatic rings of the toluene molecules have been constrained to fit regular hexagons (AFIX 66 line in SHELXL), and all of the C atoms of the toluene molecules have been restrained to isotropic behavior (ISOR line in SHELXL, s.u. 0.01). The overall quality of the crystals was rather low, leading to a final *R*_1_ factor of 0.2778.

### 4.11 Computational Details

Geometry optimizations of clusters **10**, **11**, **15a**, and **15b** were performed in the gas phase using the range-separated hybrid DFT functional ωB97X.^56^ The basis set used was the Ahlrichs’ def2 split-valence, with polarization and diffusion functions and relativistic ECP for Au.^57^ Single-point calculations on the optimized structures of **10**, **11**, **15a**, and **15b** and on the models for compound **17** were carried out at the same theoretical level, including nonlocal correlation by the VV10 functional (wB97X-v).^58^ Geometry optimizations of **3**, **8**, and **13** were carried out using the PBEh-3c method, which is a reparameterized version of PBE0 (with 42% HF exchange) that uses a split-valence double-ζ basis set (def2-mSVP) and adds three corrections that consider dispersion, basis set superposition, and other basis set incompleteness effects.^59^ The C-PCM solvation model was added to PBEh-3c calculations,^60^ considering a dielectric constant of 41.7 and a refractive index of 1.45544, intermediate between the values reported for dmso and dmf. The “restricted” approach was used in all cases. Calculations were performed with ORCA version 4.0.1.2.^61^ The output, converted in .molden format, was used for AIM and Hirshfeld analyses,^62^ performed with Multiwfn version 3.5.^63^ Cartesian coordinates of the DFT-optimized structures are collected in a separate .xyz file.

## Supporting Information Available

Crystals and experimental details (PDF)
Optimized coordinates (XYZ)

## Supplementary Material

ic9b02912_si_001.pdf

ic9b02912_si_002.xyz

## Accession Codes

CCDC 1955604–1955609 contain the supplementary crystallographic data for this paper. These data can be obtained free of charge via www.ccdc.cam.ac.uk/data_request/cif, or by emailing data_request@ccdc.cam.ac.uk, or by contacting The Cambridge Crystallographic Data Centre, 12 Union Road, Cambridge CB2 1EZ, UK; fax: +44 1223 336033.
