# Supplementary material for: Thermal Growth of Au–Fe Heterometallic Carbonyl Clusters Containing N-Heterocyclic Carbene and Phosphine Ligands
Source: Inorg Chem. 2020 Jan 31;59(4):2228–40. doi: 10.1021/acs.inorgchem.9b02912 (PMC7997394; doi:10.1021/acs.inorgchem.9b02912)
Supplement: Supplementary file 1 — ic9b02912_si_001.pdf [file ic9b02912_si_001.pdf]

Supporting Information for

Thermal Growth of Au-Fe Heterobimetallic Carbonyl Clusters Containing *N*-Heterocyclic Carbene and Phosphine Ligands

Beatrice Berti,† Marco Bortoluzzi,‡ Cristiana Cesari,† Cristina Femoni,† Maria Carmela Iapalucci,† Rita Mazzoni,† Federico Vacca,† and Stefano Zacchini*,†

† Dipartimento di Chimica Industriale "Toso Montanari", University of Bologna, Viale Risorgimento 4, I-40136 Bologna Italy. E-mail: stefano.zacchini@unibo.it; Web: https://www.unibo.it/sitoweb/stefano.zacchini/en; Tel: +39 051 2093711.

‡ Dipartimento di Scienze Molecolari e Nanosistemi, Ca' Foscari University of Venice, Via Torino 155 – 30175 Mestre (Ve), Italy.

Page/s

NMR spectra S2-S10
Figure of the molecular structure of **9** S11
Potential Solvent Accessible Voids of **17** S12-S13
Tables S14-S20
DFT-optimized structure of **10** S21
References S22

Figure S1

*^{1}H NMR spectrum of $[NEt_4][Fe_2(CO)_8(AuIPr)]$ ($[NEt_4]$[**11**]) in CD_3COCD_3 at 298 K.*

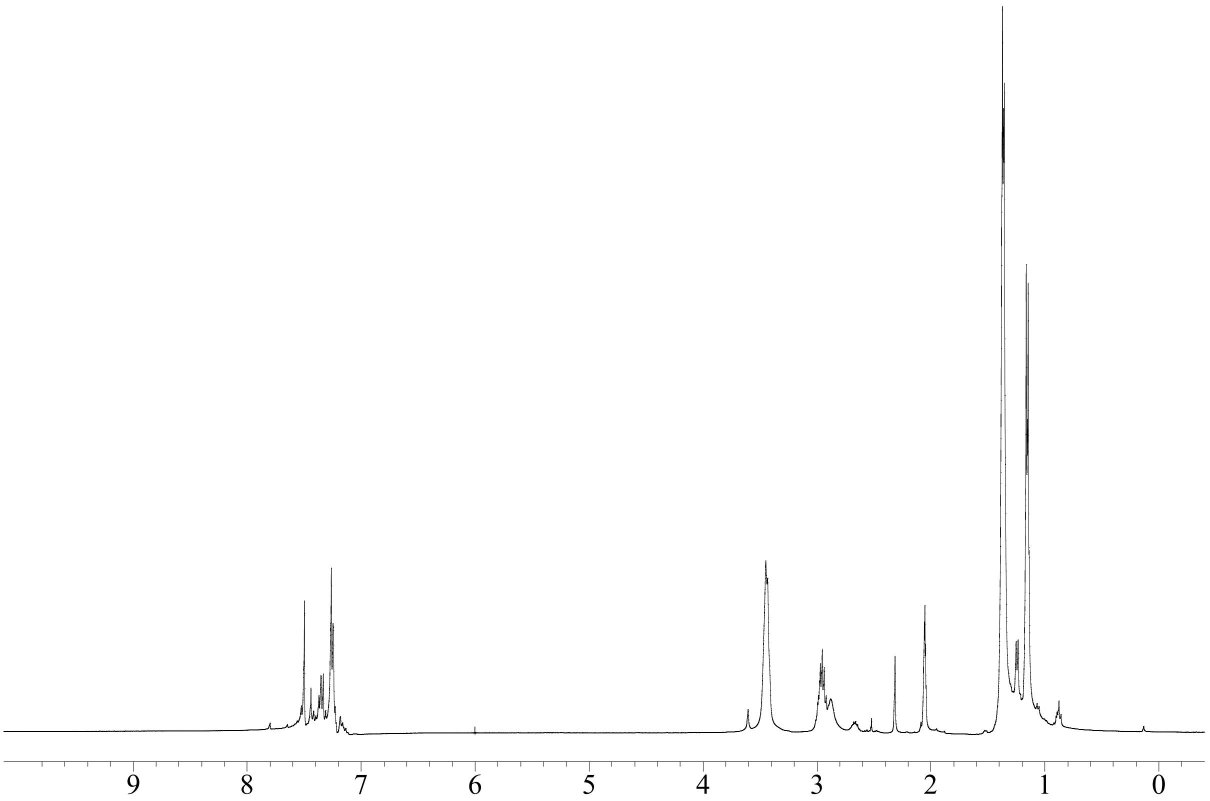

Figure S2

*$^{13}C\{^{1}H\}$ NMR spectrum of $[NEt_4][Fe_2(CO)_8(AuIPr)]$ ($[NEt_4]$[**11**]) in CD_3COCD_3 at 298 K*

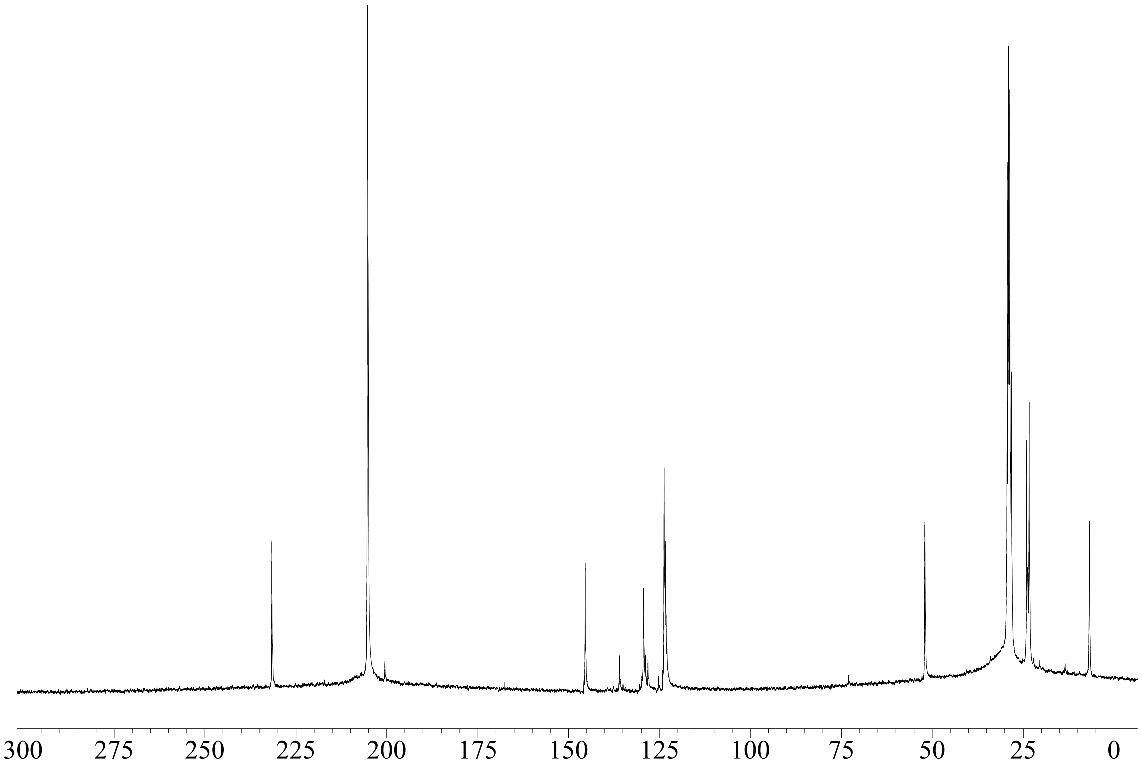

Figure S3

*$^{13}C\{^{1}H\}$ NMR spectra in the carbonyl region of $[NEt_4][Fe_2(CO)_8(AuIPr)$ ($[NEt_4][$**11**$])]$ in CD_3COCD_3 at 273 K and 213 K.*

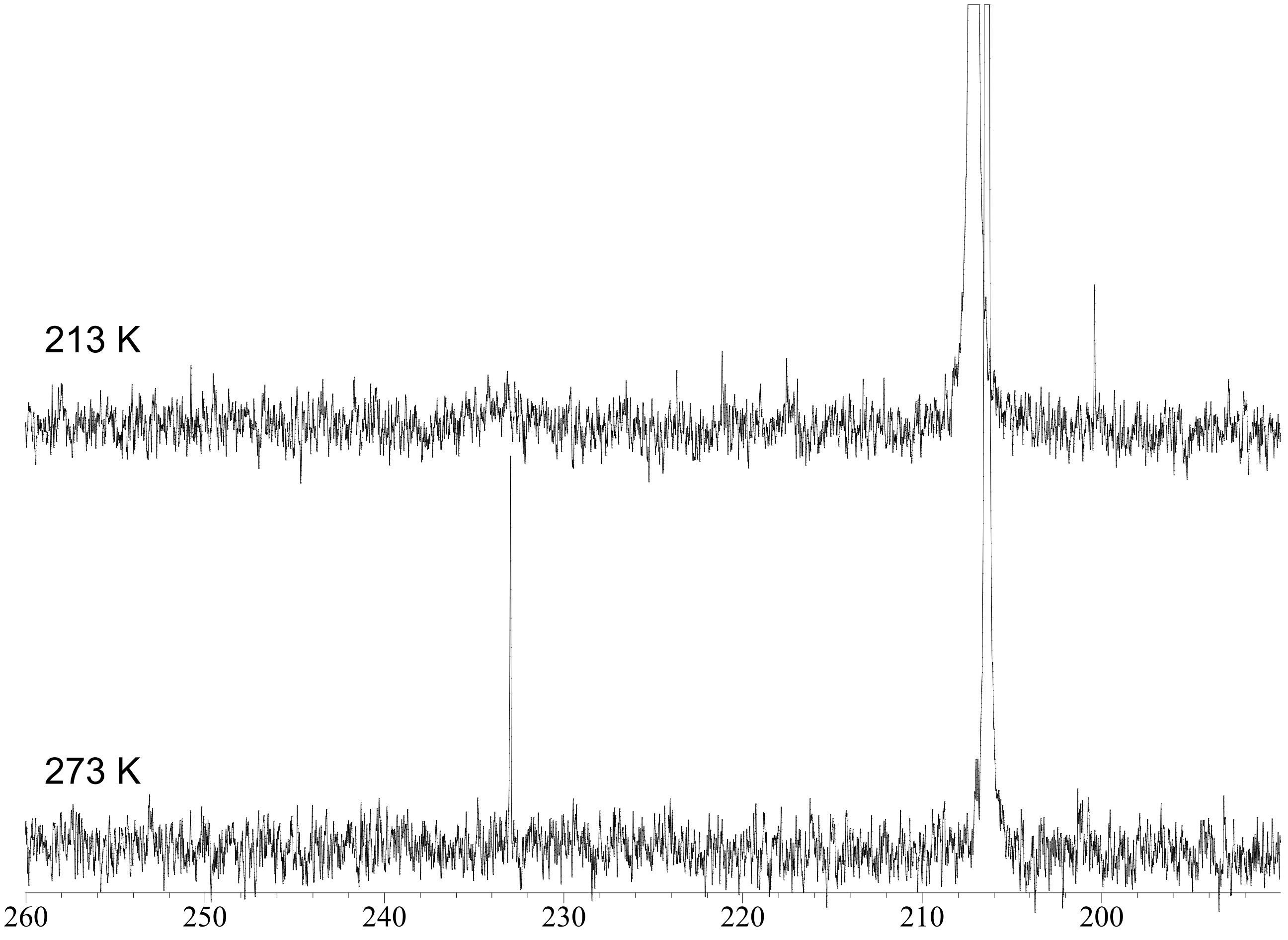

Figure S4

*^{1}H NMR spectrum of $[NBu_4][Au_3Fe_2(CO)_8(IMes)_2]$ ($[NBu_4]$[**13**]) in CD_2Cl_2 at 298 K.*[2]

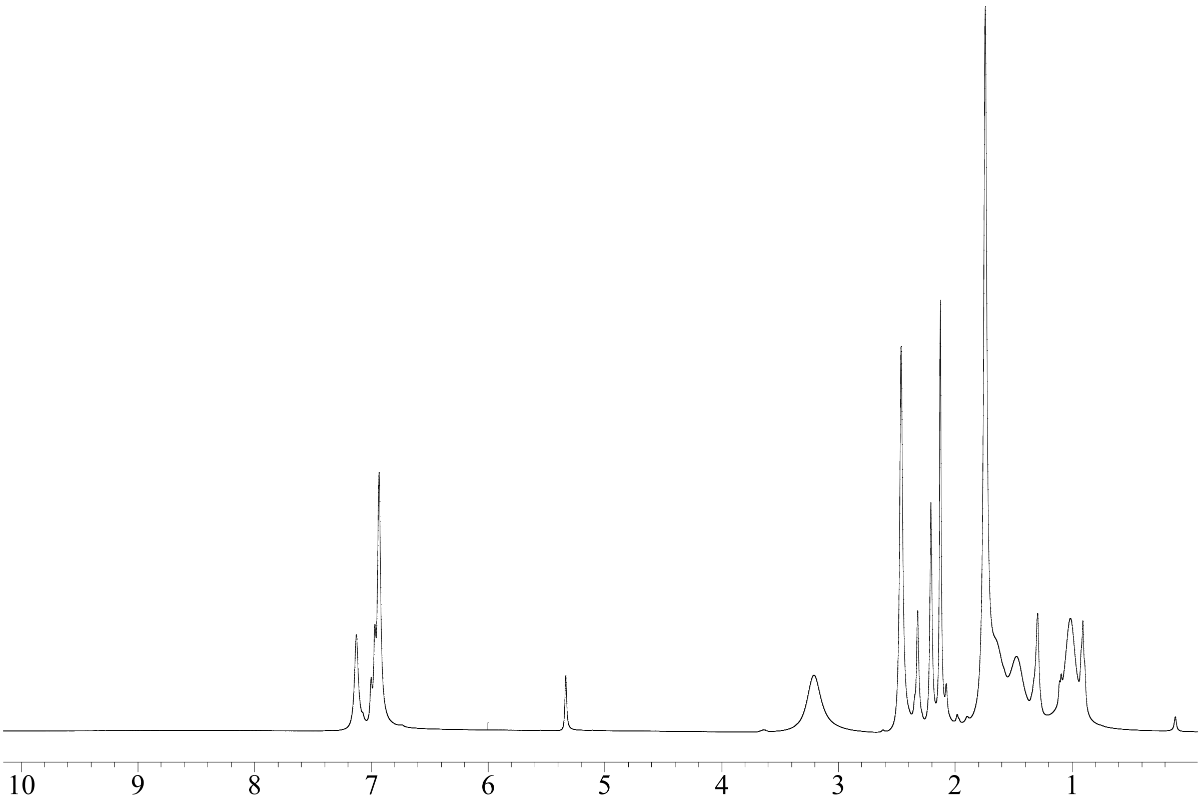

Figure S5

*^{1}H NMR spectrum of $[NBu_4][Au_3Fe_2(CO)_8(IMes)_2]$ ($[NBu_4]$[**13**]) in CD_2Cl_2 at 298 K.*[2]

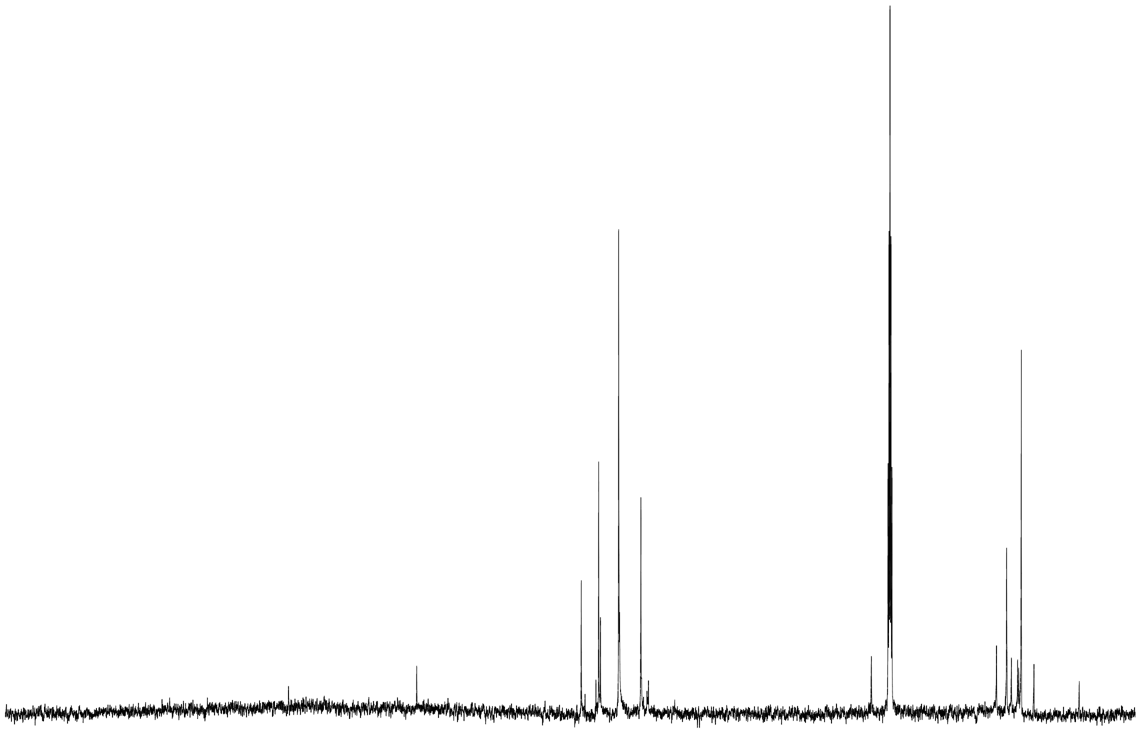

Figure S6

*^{1}H NMR spectrum of $[NMe_4]_2[Au(IMes)_2][Au_3\{Fe(CO)_4\}_3]$ ($[NMe_4]_2[Au(IMes)_2]$[**8**]) in CD_3CN at 298 K.*[1]

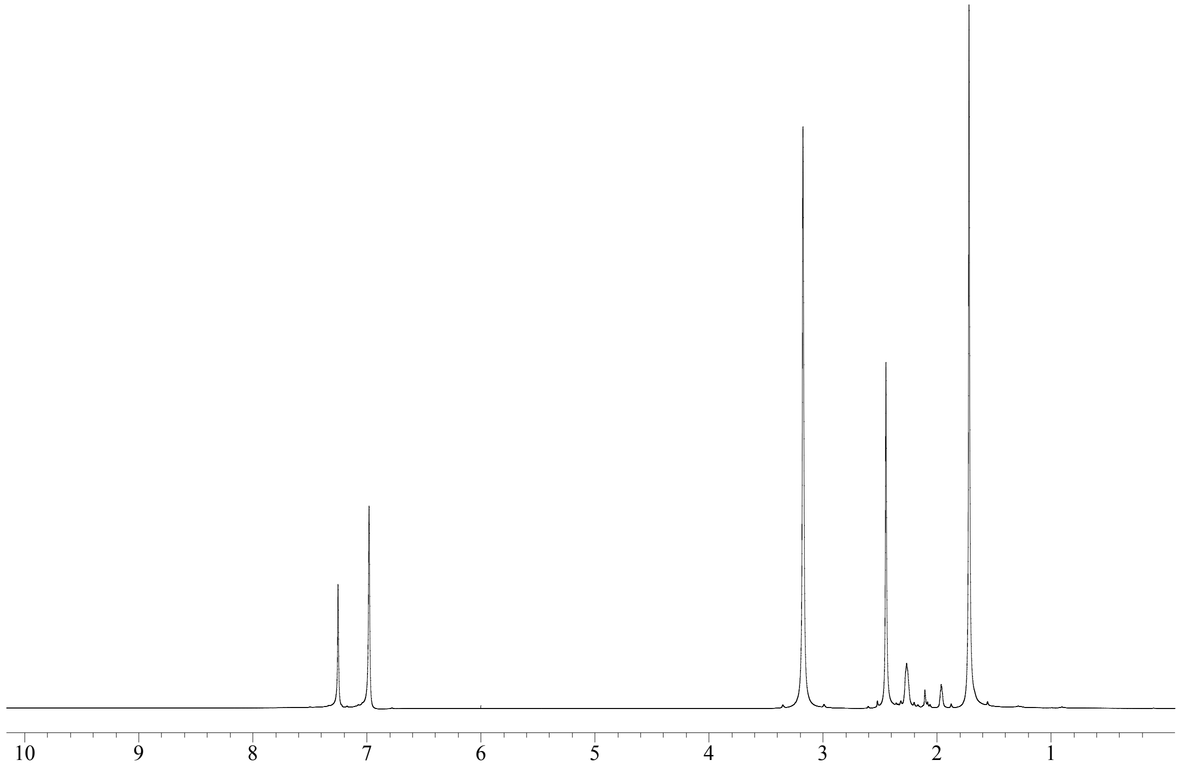

Figure S7

*$^{13}C\{^{1}H\}$ NMR spectrum of $[NMe_4]_2[Au(IMes)_2][Au_3\{Fe(CO)_4\}_3]$ ($[NMe_4]_2[Au(IMes)_2]$[**8**]) in CD_3CN at 298 K.*[1]

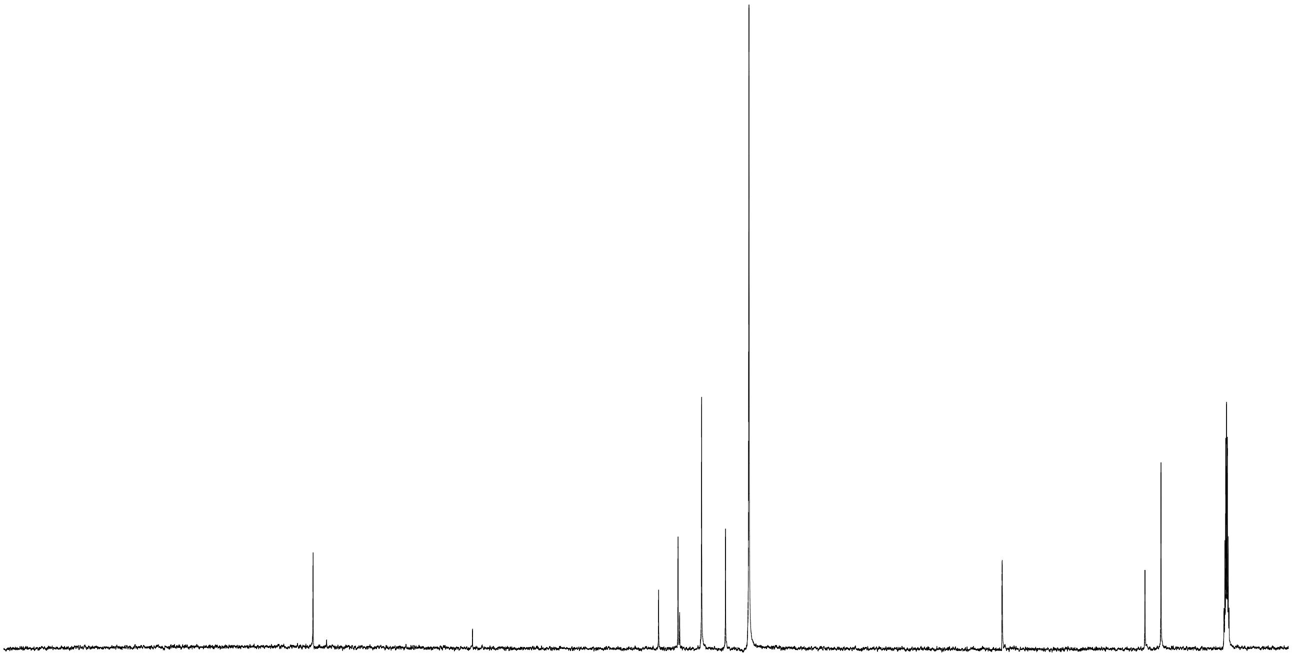

Figure S8

*$^{31}P\{^1H\}$ NMR spectrum of $Fe(CO)_4(AuIMes)(AuPPh_3)$ (**6**) in CD_3COCD_3 at 298 K.*[3]

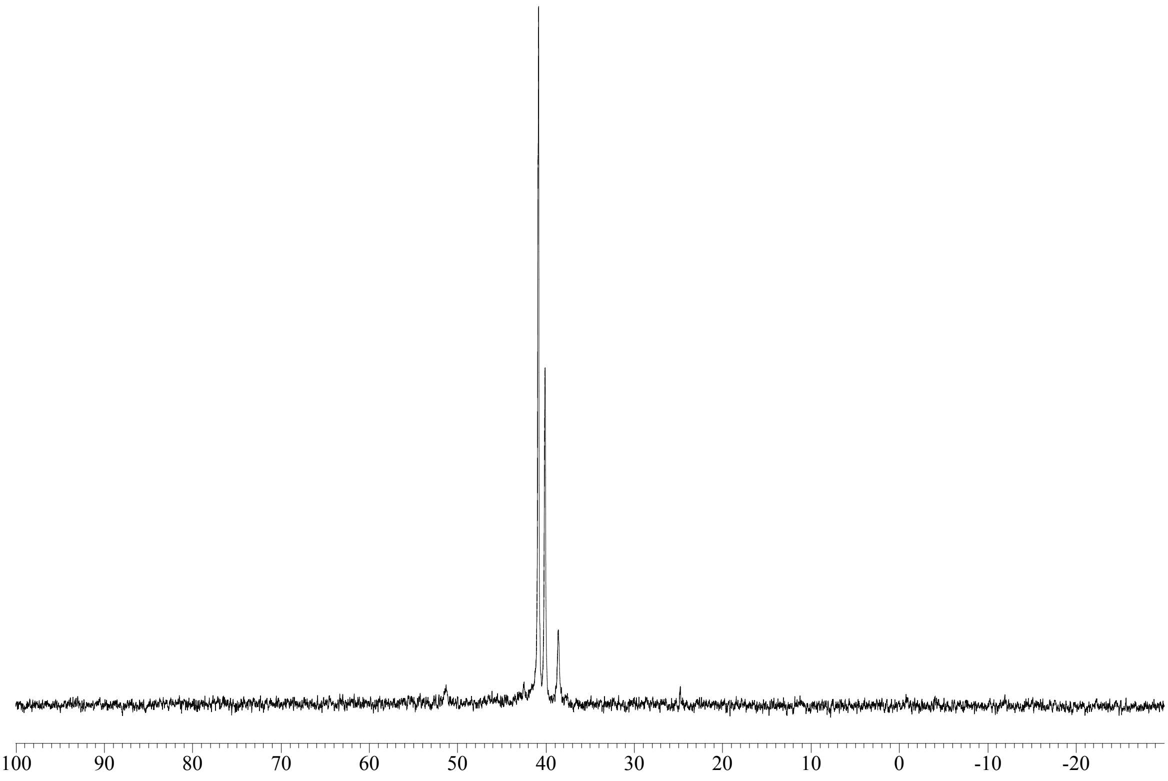

Figure S9

*$^{31}P\{^1H\}$ NMR spectra in CD_3COCD_3 at 298 K of (a) $Fe(CO)_4(AuIMes)(AuPPh_3)$ (**6**) and (b) the mixture obtained after thermal treatment of **6**. The three resonances at δ_P 40.8, 40.1 and 38.5 ppm in (a) and (b) corresponded to **6**, **14** and **15**, respectively.*

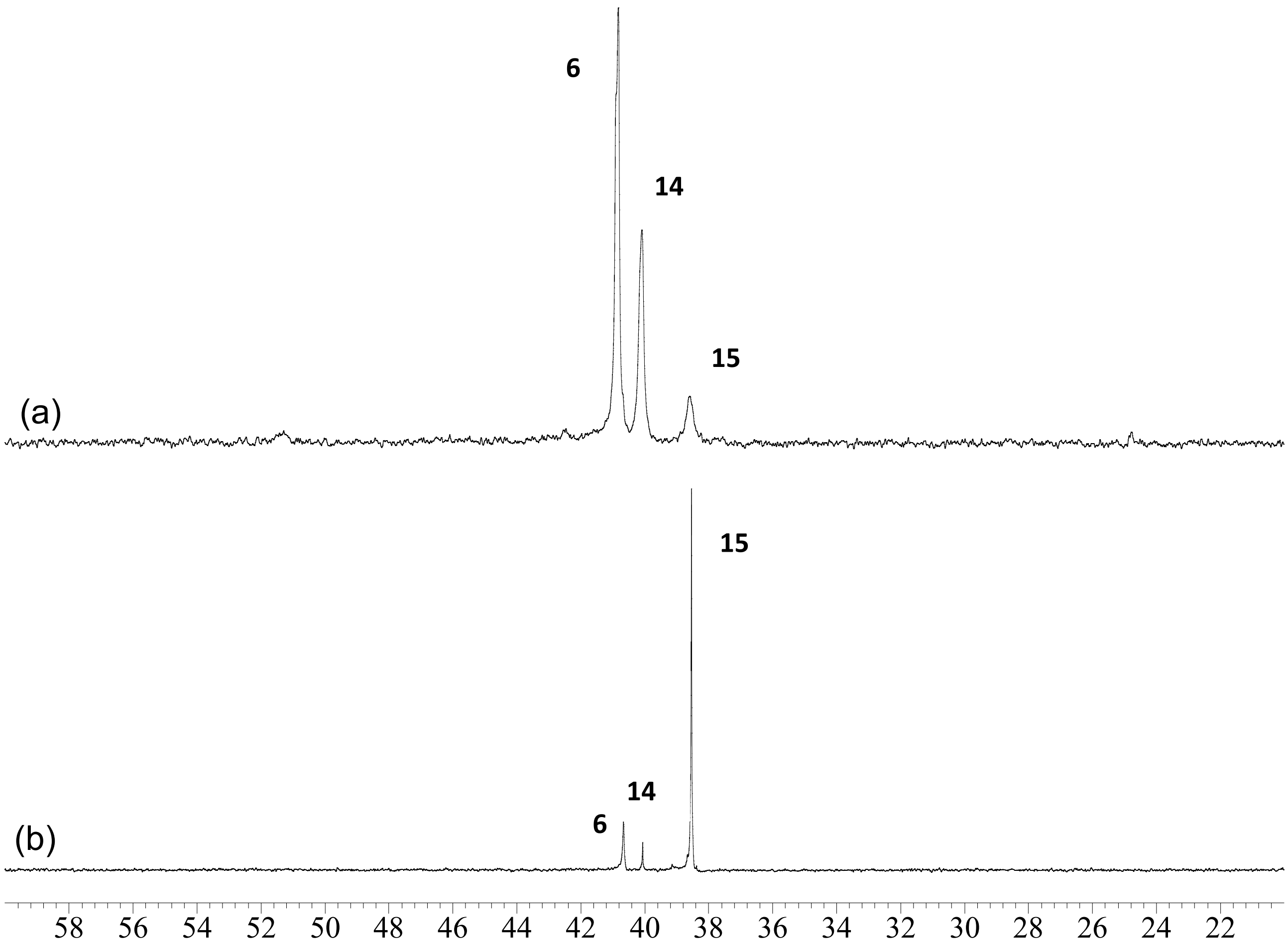

Figure S10

*$^{31}P\{^{1}H\}$ NMR spectrum of $[Au(IMes)_2][Au_3\{Fe(CO)_4\}_2(PPh_3)_2]$ ($[Au(IMes)_2]$[**15**]) in CD_3COCD_3 at 298 K.*

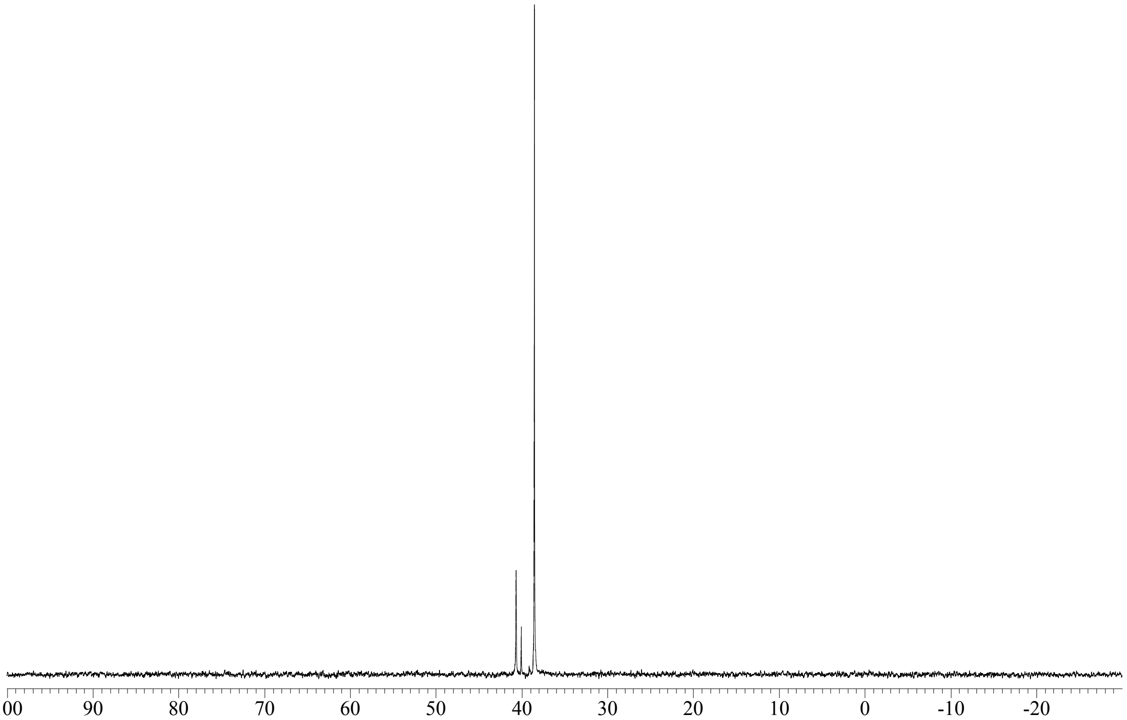

Figure S11

*^{1}H NMR spectrum of $[Au(IMes)_2][Au_3\{Fe(CO)_4\}_2(PPh_3)_2]$ ($[Au(IMes)_2]$[**15**]) in CD_3COCD_3 at 298 K.*

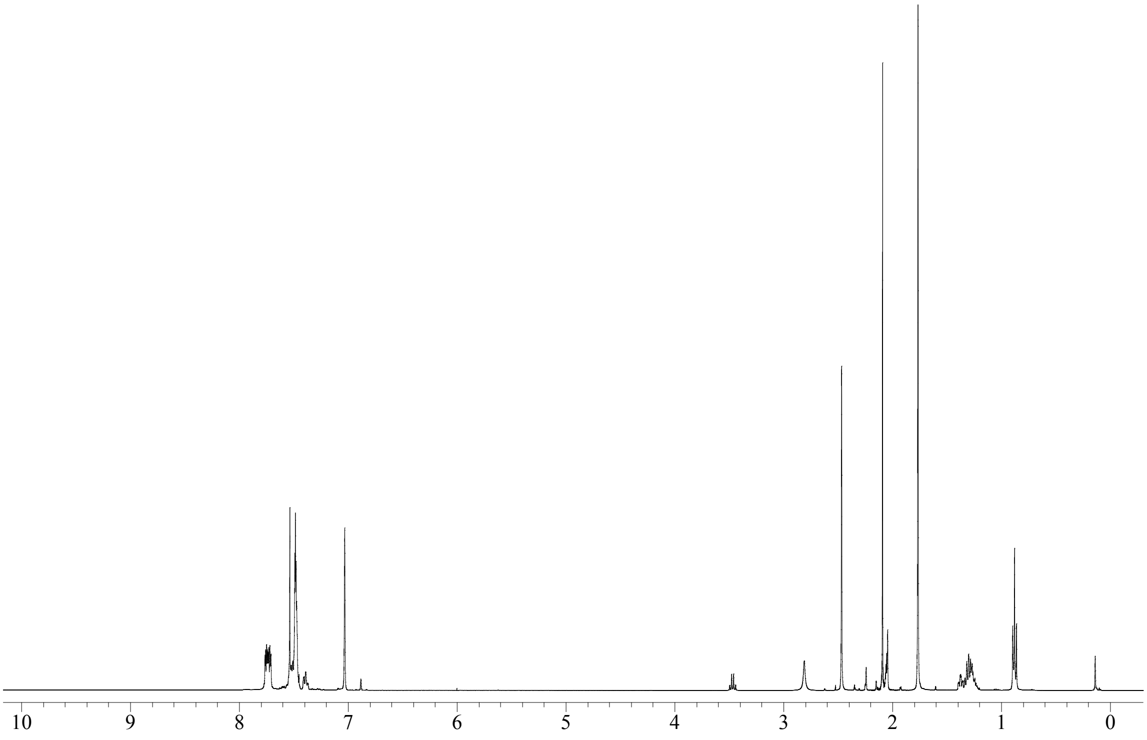

Figure S12

*$^{13}C\{^1H\}$ NMR spectrum of $[Au(IMes)_2][Au_3\{Fe(CO)_4\}_2(PPh_3)_2]$ ($[Au(IMes)_2]$[**15**]) in CD_3COCD_3 at 298 K.*

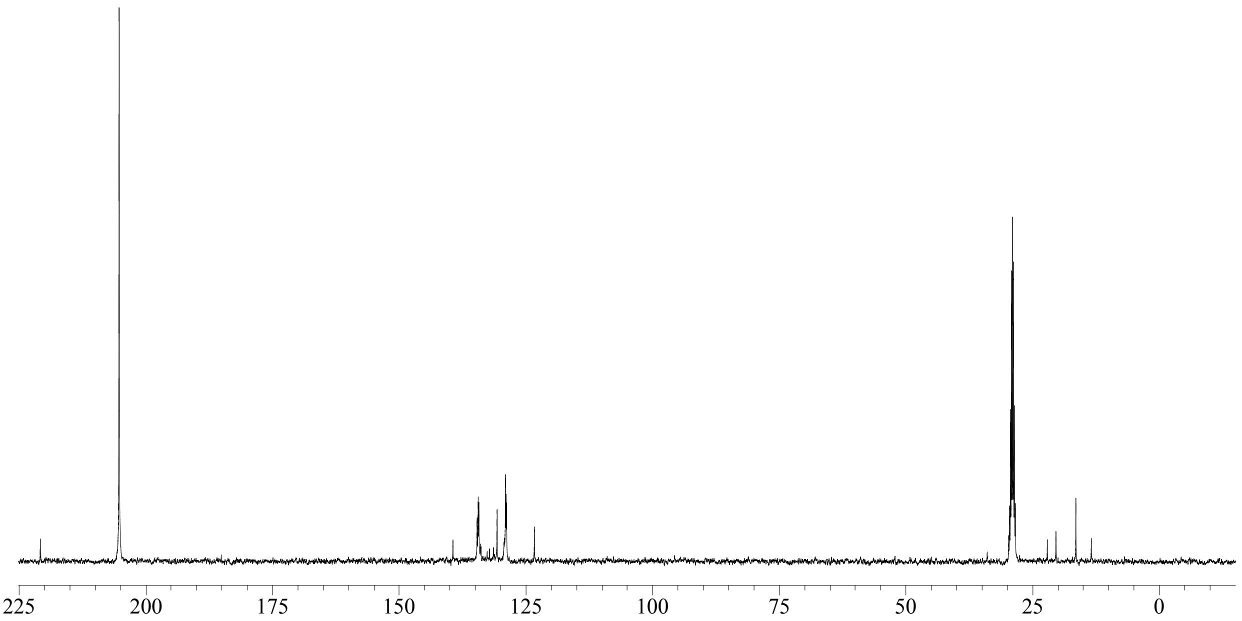

Figure S13

*^1H NMR spectrum of $[Au_{16}S\{Fe(CO)_4\}_4(IPr)_4]^{n+}$ (**17**) in CD_3COCD_3 at 298 K.*

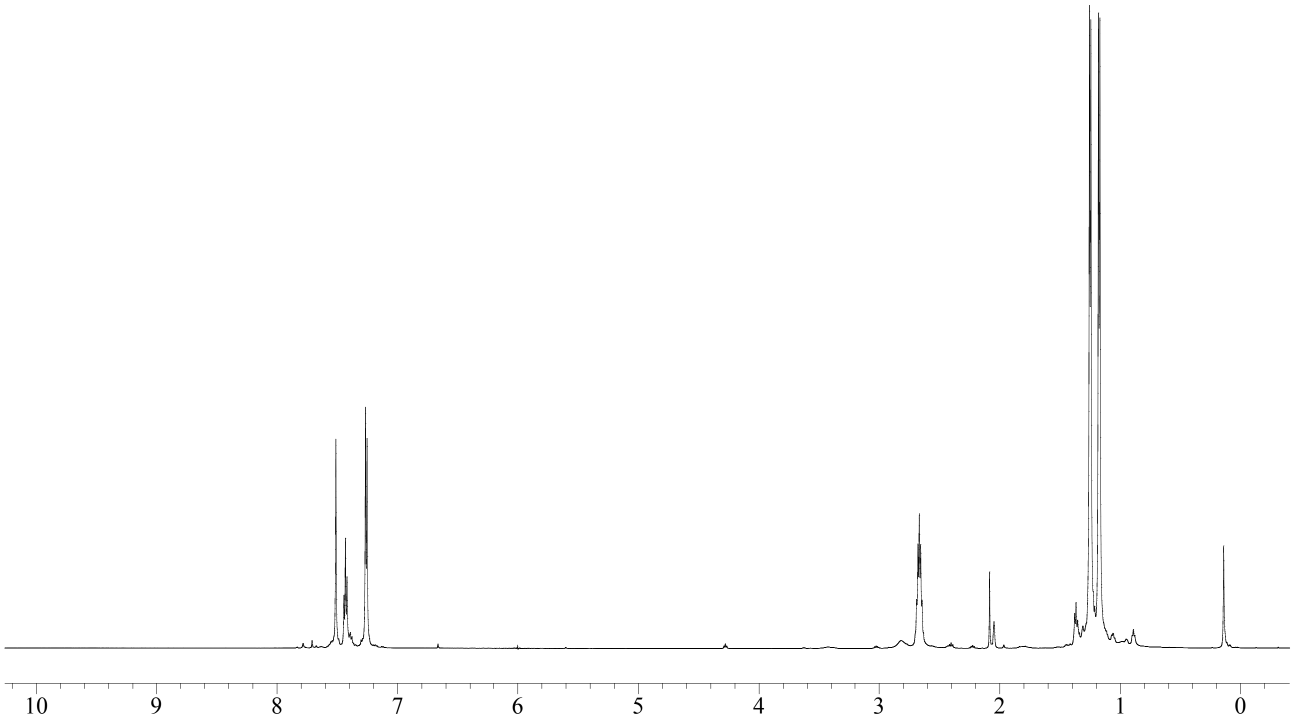

Figure S14

*$^{13}C\{^{1}H\}$ NMR spectrum of $[Au_{16}S\{Fe(CO)_4\}_4(IPr)_4]^+$ (**17**) in CD_3COCD_3 at 298 K.*

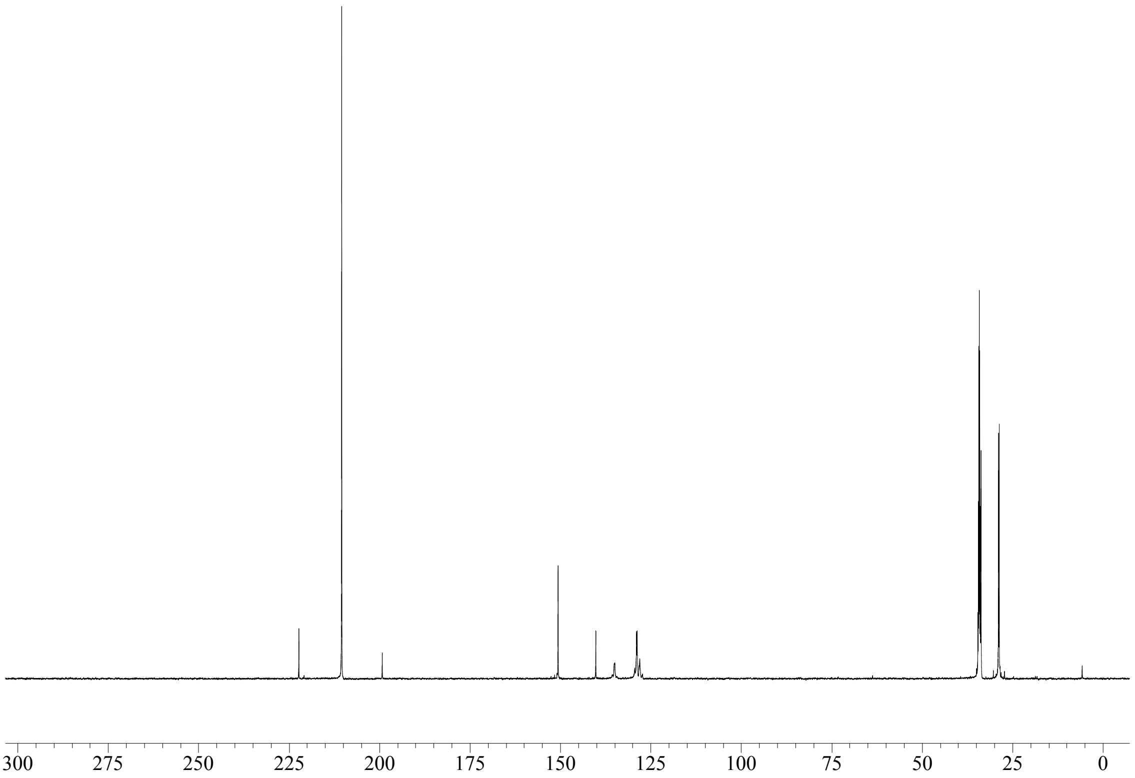

Figure S15

*^{19}F NMR spectrum of $[Au_{16}S\{Fe(CO)_4\}_4(IPr)_4]^+$ (**17**) in CD_3COCD_3 at 298 K, which shows the typical resonances of $[BF_4]^-$*

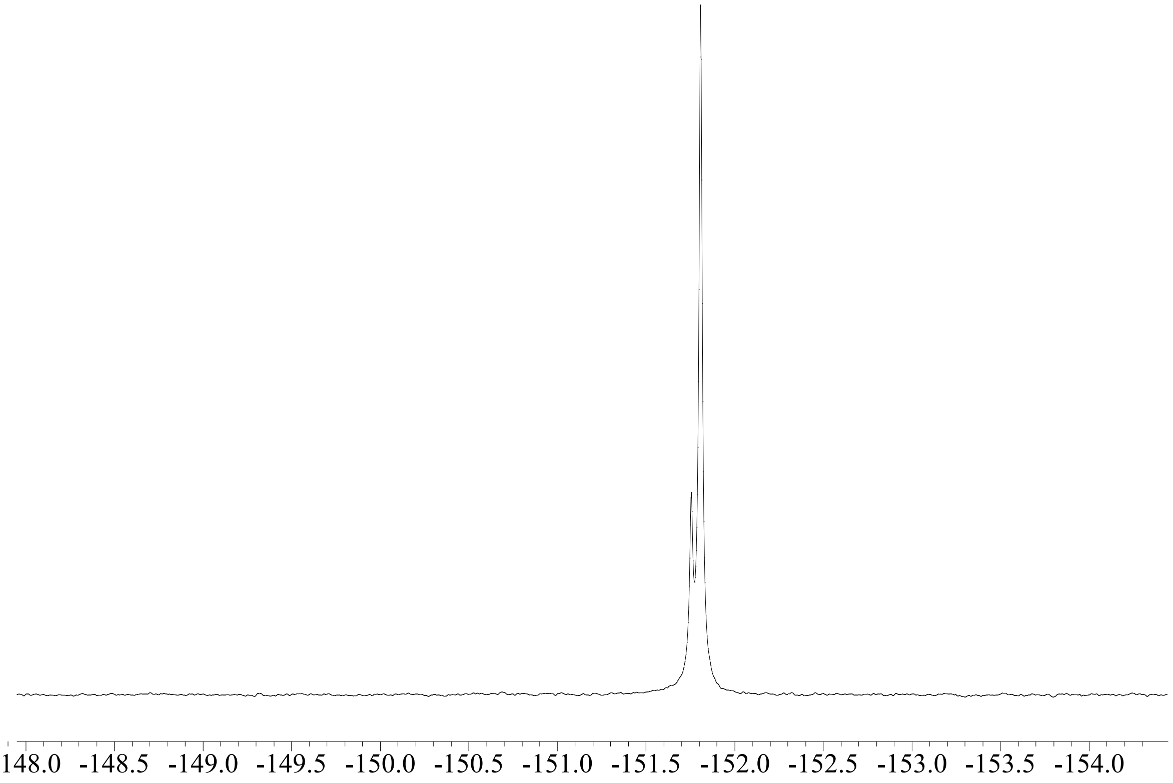

Figure S16

Molecular structure of $[Fe_3S(CO)_9]^{2-}$ (**9**). (green Fe; orange S; red O; grey C)

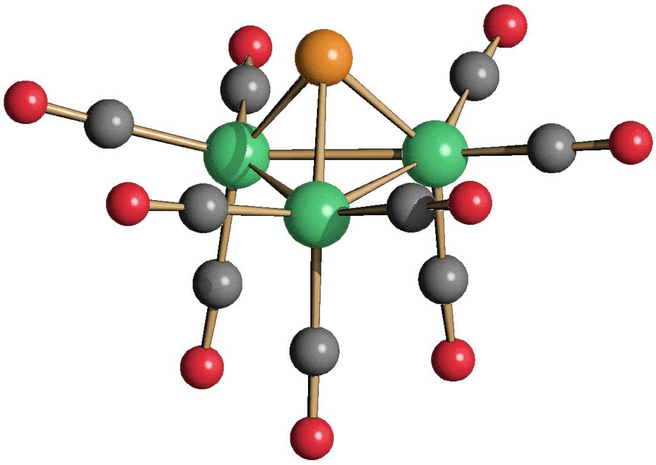

Figure S17

Views of the potential solvent accessible voids present within the crystal packing of $[Au_{16}S\{Fe(CO)_4\}_4(IPr)_4]^{n+}$ *(**17**) along the crystallographic axes a, b, and c.*

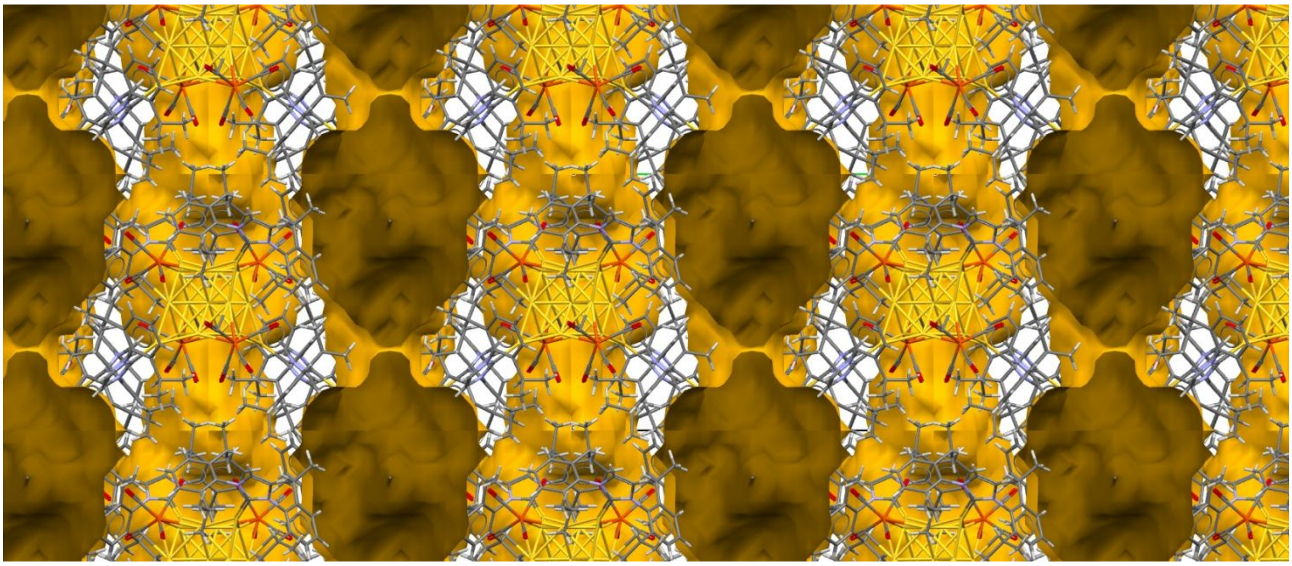

(a)

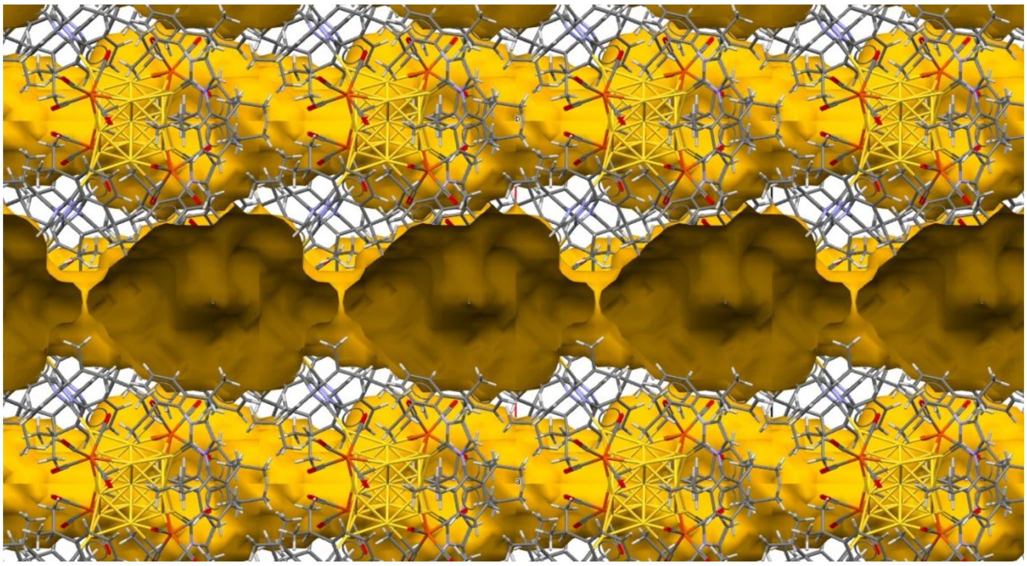

(b)

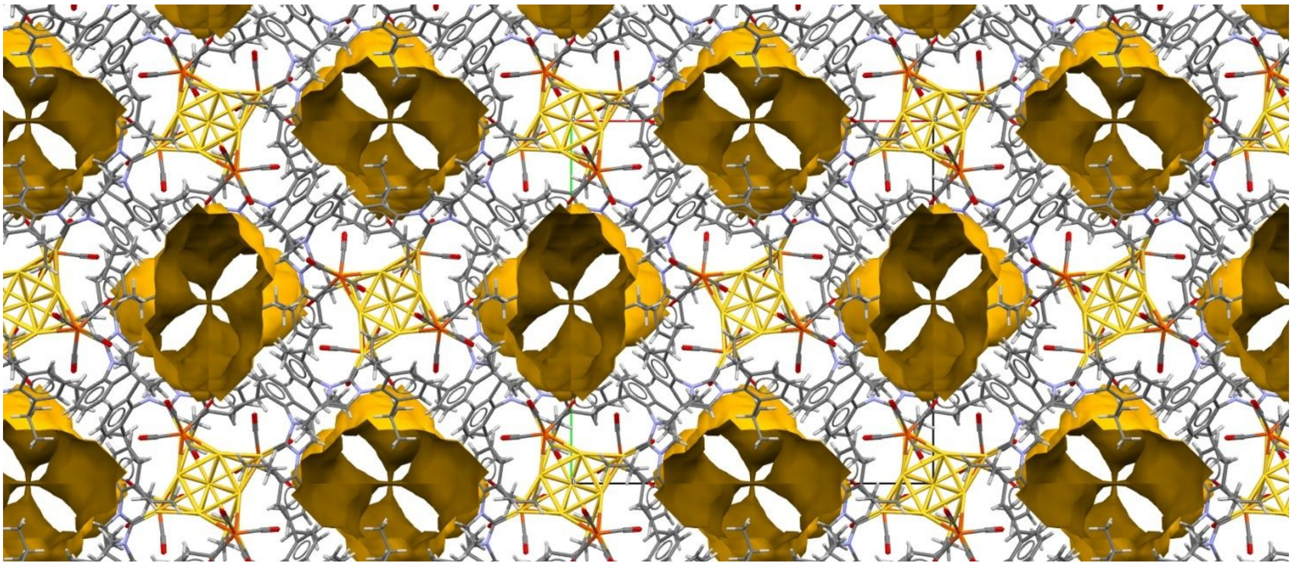

(c)

Table S1

*Main bond distances (Å) and angles (deg) of the two isomers of $[Au_3Fe_2(CO)_8(PPh_3)_2]^-$ (**15**).*

	**15a**	**15b***
Au(1)-Fe(1)	2.529(3)	2.547(9)
Au(2)-Fe(1)	2.560(4)	2.601(11)
Au(2)-Fe(2)	2.586(4)	2.601(11)
Au(3)-Fe(2)	2.538(3)	2.547(9)
Au(1)-Au(2)	2.9353(15)	2.9177(14)
Au(2)-Au(3)	2.8855(14)	2.9177(14)
Au(1)-P(1)	2.259(6)	2.271(10)
Au(3)-P(3)	2.278(6)	2.271(10)
Fe(1)-Au(1)-P(1)	174.02(19)	173.0(3)
Fe(2)-Au(3)-P(3)	172.96(18)	173.0(3)
Fe(1)-Au(2)-Fe(2)	170.01(12)	180.0
Au(1)-Fe(1)-Au(2)	70.45(9)	69.0(3)
Au(2)-Fe(2)-Au(3)	68.53(9)	69.0(3)
Fe(1)-Au(1)-Au(2)	55.27(9)	56.4(2)
Fe(2)-Au(3)-Au(2)	56.53(9)	56.4(2)
Fe(1)-Au(2)-Au(1)	133.12(9)	54.59(16)
Fe(2)-Au(2)-Au(3)	54.94(8)	54.59(16)
Au(1)-Au(2)-Au(3)	132.00(4)	180.00(10)

* Au(2) in **15b** is located on an inversion centre making Au(1)/Au(3) and Fe(1)/Fe(3) equivalent.

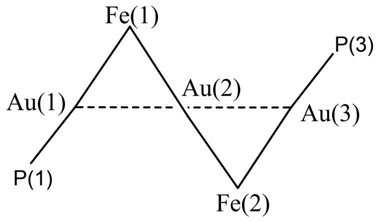

Table S2

*Properties of the Fe-Au (3,-1) b.c.p in **10**, **11**, **15a** and **15b** (electron density, ρ; potential energy density, V; energy density, E; Laplacian of electron density, $\nabla^2 \rho$). All the quantities were reported in a.u.*

Compound	Bond	ρ	V	E	$\nabla^2 \rho$
**10**	Fe-Au	0.047	-0.039	-0.013	0.054
**11**	Fe-Au	0.046	-0.038	-0.012	0.054
**15a**	Fe-$Au_{central}$	0.050	-0.043	-0.014	0.064
	Fe-$Au_{terminal}$	0.060	-0.053	-0.017	0.077
**15b**	Fe-$Au_{central}$	0.049	-0.043	-0.013	0.064
	Fe-$Au_{terminal}$	0.060	-0.052	-0.017	0.076

Table S3

*Main bond distances (Å) and angles (deg) of $[Au_{16}S\{Fe(CO)_4\}_4(IPr)_4]^{n+}$ (**17**).*

	**Range**	**Average**
Au_c-Au_c	2.702(2)-2.874(2)	2.753(6)
Au_c-Au_s	2.724(2)-2.733(2)	2.728(3)
Au_c-Fe	2.625(5)-2.650(5)	2.636(9)
Au_c-S	2.7641(13)-2.7995(16)	2.777(3)
Au_s-$N_{carbene}$	2.04(4)	2.04(4)
Au_c···C(O)	2.636(4)-2.723(4)	2.684(10)

Au_c = Au atoms of the Au_{12} cube-octahedron

Au_s = Au atoms of the AuIPr fragments.

Table S4

Properties of the (3,-1) b.c.p. involving Au centres in the model compound $[Au_{16}S\{Fe(CO)_4\}_4(NHC)_4]^{2+}$ (NHC = 1,3-dimethylimidazol-2-ylidene). Electron density, ρ; potential energy density, V; energy density, E; Laplacian of electron density, $\nabla^2\rho$. All the quantities are reported in a.u.

	Bond	ρ	V	E	$\nabla^2\rho$
**(a)**	Au-S	0.042	-0.034	-0.005	0.099
**(b)**	Au-Fe	0.052	-0.047	-0.014	0.077
**(c)**	Au-Au	0.043	-0.042	-0.008	0.106
**(d)**	Au-Au	0.057	-0.062	-0.015	0.123
**(e)**	Au-Au	0.055	-0.056	-0.014	0.112
**(f)**	Au-NHC	0.139	-0.193	-0.068	0.233

Table S5

Crystal data and experimental details for $[Au(IMes)_2][Au_3\{Fe(CO)_4\}_2(PPh_3)_2]\cdot0.67CH_2Cl_2$, $[Au(IPr)_2][HFe(CO)_4]$, $[NEt_4]_2[Fe_3S(CO)_9]$, $[NEt_4][Fe_3(CO)_{10}(CCH_3)]$, $[Au_{16}S\{Fe(CO)_4\}_4(IPr)_4][BF_4]_n\cdot$solv, and $[NEt_4][Fe_2(CO)_8(AuIPr)]\cdot1.5$toluene

	**$[Au(IMes)_2]$ $[Au_3\{Fe(CO)_4\}_2(PPh_3)_2]\cdot$ $0.67CH_2Cl_2$,**	**$[Au(IPr)_2]$ $[HFe(CO)_4]$**	**$[NEt_4]_2[Fe_3S(CO)_9]$**
Formula	$C_{86.67}H_{79.33}Au_4Cl_{1.33}Fe_2N_4O_8P_2$	$C_{58}H_{72}AuFeN_4O_4$	$C_{25}H_{40}Fe_3N_2O_9S$
Fw	2313.64	1142.01	712.20
T, K	100(2)	100(2)	100(2)
λ, Å	0.71073	0.71073	0.71073
Crystal system	Triclinic	Monoclinic	Monoclinic
Space Group	$P\bar{1}$	*C2/c*	*Pc*
a, Å	11.9307(8)	16.9272(10)	14.1253(10)
b, Å	23.7175(17)	17.9331(10)	11.3116(8)
c, Å	24.8265(17)	18.0055(11)	19.4702(13)
α, °	116.667(2)	90	90
β, °	95.568(2)	97.405(2)	90.966(2)
γ, °	92.779(2)	90	90
Cell Volume, $Å^3$	6214.6(8)	5420.1(6)	3110.5(4)
Z	3	4	4
D_c, g cm^{-3}	1.855	1.399	1.521
μ, mm^{-1}	7.535	3.019	1.498

F(000)	3336	2340	1480
Crystal size, mm	0.18×0.15×0.12	0.16×0.13×0.11	0.16×0.12×0.10
θ limits, °	1.615–25.100	1.662–25.668	1.800–25.995
Reflections collected	74357	29792	39325
Independent reflections	22142 [R_{int} = 0.1330]	5117 [R_{int} = 0.1077]	12171 [R_{int} = 0.0799]
Completeness to θ max	99.9%	100.0%	100.0%
Data / restraints / parameters	22124 / 2218 / 1392	5117 / 279 / 339	12171 / 842 / 838
Goodness on fit on F^2	1.135	1.048	1.034
R_1 (I > 2σ(I))	0.1276	0.0491	0.0592
wR_2 (all data)	0.2534	0.0652	0.1056
Largest diff. peak and hole, e Å^{-3}	6.357 / -7.695	1.741 / -2.168	0.619 / -0.716

	**$[NEt_4][Fe_3(CO)_{10}(CCH_3)]$**	**$[Au_{16}S\{Fe(CO)_4\}_4(IPr)_4][BF_4]_n$·solv**	**$[NEt_4][Fe_2(CO)_8(AuIPr)]$·1.5toluene**
Formula	$C_{20}H_{23}Fe_3NO_{10}$	$C_{124}H_{144}Au_{16}Fe_4N_8O_{16}S$	$C_{53.5}H_{68}AuFe_2N_3O_8$
Fw	604.94	5409.39	1189.77
T, K	100(2)	100(2)	100(2)

λ, Å	0.71073	0.71073	0.71073
Crystal system	Monoclinic	Tetragonal	Monoclinic
Space Group	$P2_1/n$	$P\bar{4}2_1c$	$P2_1/c$
a, Å	14.3389(5)	22.4854(5)	19.541(2)
b, Å	12.0119(5)	22.4854(5)	15.5847(16)
c, Å	14.7414(6)	15.9065(4)	17.4146(19)
α, °	90	90	90
β, °	109.1130(10)	90	95.924(3)
γ, °	90	90	90
Cell Volume, $Å^3$	2399.06(16)	8042.2(4)	5275.0(10)
Z	4	2	4
D_c, g cm^{-3}	1.675	2.234	1.498
μ, mm^{-1}	1.844	14.938	3.371
F(000)	1232	4912	2420
Crystal size, mm	0.19×0.16×0.14	0.14×0.12×0.10	0.19×0.16×0.12
θ limits, °	1.720–25.050	1.568–25.092	1.048–24.998
Reflections collected	28656	115672	52756
Independent	4249 [R_{int} = 0.0344]	7150 [R_{int} = 0.1949]	9230 [R_{int} = 0.1023]

reflections			
Completeness to θ max	100.0%	100.0%	99.2%
Data / restraints / parameters	4249 / 6 / 312	7150 / 37 / 358	9230 / 516 / 616
Goodness on fit on F^2	1.062	1.107	1.232
R_1 ($I > 2\sigma(I)$)	0.0296	0.0514	0.1235
wR_2 (all data)	0.0640	0.1799	0.2778
Largest diff. peak and hole, e $Å^{-3}$	1.230 / -0.419	2.538 / -2.124	6.990 / -5.097

Figure S18

*DFT-optimized geometry of **10**. Selected computed bond lengths (Å): Fe-Fe 2.588; Fe-Au 2.693, 2.704; Fe-CO(terminal) 1.759, 1.789, 1.790, 1.759, 1.789, 1.790; Fe-CO(bridging) 1.960, 1.961, 1.963, 1.963; Au-C 2.059. Colour map: Au, yellow; Fe, green; O, red; N, blue; C, grey. Hydrogen atoms are omitted for clarity.*

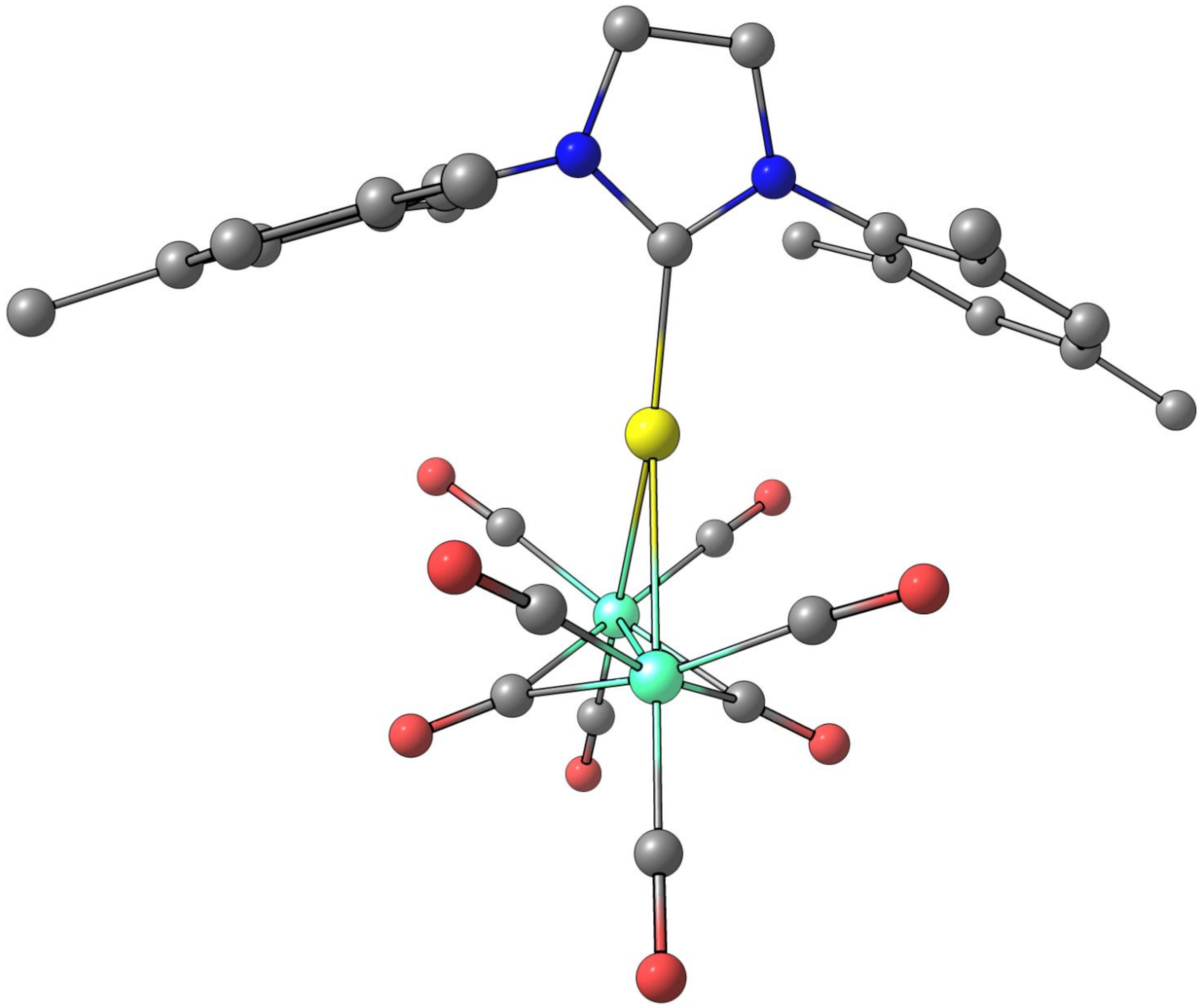

References

(1) Berti, B.; Bortoluzzi, M.; Cesari, C.; Femoni, C.; Iapalucci, M. C.; Mazzoni, R.; Vacca, F.; Zacchini, S. *Inorg. Chem.* Polymerization Isomerism in $[\{MFe(CO)_4\}_n]^{n-}$ (M = Cu, Ag, Au; N = 3, 4) Molecular Cluster Supported by Metallophilic Interactions. **2019**, *58*, 2911-2915.

(2) Bortoluzzi, M.; Cesari, C.; Ciabatti, I.; Femoni, C.; Hayatifar, M.; Iapalucci, M. C.; Mazzoni, R.; Zacchini, S. Bimetallic FeAu Carbonyl Clusters Derived from Collman's Reagent: Synthesis, Structure and DFT Analysis of $Fe(CO)_4(AuNHC)_2$ and $[Au_3Fe_2(CO)_8(NHC)_2]^-$. *J. Clust. Sci.* **2017**, *28*, 703-723.

(3) Berti, B.; Bortoluzzi, M.; Cesari, C.; Femoni, C.; Iapalucci, M. C.; Mazzoni, R.; Vacca, F.; Zacchini, S. Synthesis and Characterization of Heterobimetallic Carbonyl Clusters with Direct Au-Fe and Au···Au Interactions supported by *N*-Heterocyclic Carbene and Phosphine Ligands. *Eur. J. Inorg. Chem.* **2019**, 3084-3093.
